# Yeast Chaperone Hsp70-Ssb Modulates a Variety of Protein-Based Heritable Elements

**DOI:** 10.3390/ijms24108660

**Published:** 2023-05-12

**Authors:** Lina M. Jay-Garcia, Joseph L. Cornell, Rebecca L. Howie, Quincy L. Faber, Abigail Salas, Tatiana A. Chernova, Yury O. Chernoff

**Affiliations:** 1 School of Biological Sciences, Georgia Institute of Technology, Atlanta, GA 30332, USA; uai9@cdc.gov (L.M.J.-G.); jcornell9@gatech.edu (J.L.C.); fvu8@cdc.gov (R.L.H.); quincy.faber1@gmail.com (Q.L.F.); abbeysalas@gatech.edu (A.S.); 2 Department of Biochemistry, Emory University School of Medicine, Atlanta, GA 30322, USA; tcherno@emory.edu

**Keywords:** amyloid, Gγ, heat shock, Lsb2, mnemon, prion, RAC, Ssb, Ure2, yeast

## Abstract

Prions are transmissible self-perpetuating protein isoforms associated with diseases and heritable traits. Yeast prions and non-transmissible protein aggregates (mnemons) are frequently based on cross-β ordered fibrous aggregates (amyloids). The formation and propagation of yeast prions are controlled by chaperone machinery. Ribosome-associated chaperone Hsp70-Ssb is known (and confirmed here) to modulate formation and propagation of the prion form of the Sup35 protein [*PSI*^+^]. Our new data show that both formation and mitotic transmission of the stress-inducible prion form of the Lsb2 protein ([*LSB*^+^]) are also significantly increased in the absence of Ssb. Notably, heat stress leads to a massive accumulation of [*LSB*^+^] cells in the absence of Ssb, implicating Ssb as a major downregulator of the [*LSB*^+^]-dependent memory of stress. Moreover, the aggregated form of Gγ subunit Ste18, [*STE*^+^], behaving as a non-heritable mnemon in the wild-type strain, is generated more efficiently and becomes heritable in the absence of Ssb. Lack of Ssb also facilitates mitotic transmission, while lack of the Ssb cochaperone Hsp40-Zuo1 facilitates both spontaneous formation and mitotic transmission of the Ure2 prion, [*URE3*]. These results demonstrate that Ssb is a general modulator of cytosolic amyloid aggregation, whose effect is not restricted only to [*PSI*^+^].

## 1. Introduction

Mammalian prions are transmissible infectious protein isoforms that cause transmissible spongiform encephalopathies (TSEs) and are based on self-perpetuating cross-β fibrous aggregates (amyloids) [1]. Amyloids associated with devastating human disorders, such as Alzheimer’s or Parkinson’s disease, also possess certain prion features [2,3]. Yeast *Saccharomyces cerevisiae* contains a variety of endogenous prions that are heritable in a non-Mendelian fashion and frequently control detectable phenotypic traits [4,5]. Therefore, yeast prions represent a protein-based pathway of inheritance that is based on templated structures rather than on sequences. The majority of known yeast prions are amyloids, thus providing an excellent model for studying the biological and pathological aspects of amyloid formation [6]. Some prions are lethal or highly pathogenic in yeast [7,8], while other prions have been proposed to play adaptive roles [9]. [*PSI*^+^] and [*URE3*] are the best characterized yeast prions, based on amyloid forms of the translation termination factor Sup35 (eRF3) and regulatory protein in nitrogen uptake, Ure2, respectively [4,5]. The stress-inducible short-living actin-associated yeast protein Lsb2 can also form a metastable prion, [*LSB*^+^], that is inducible by heat treatment and is maintained in a fraction of the cell population recovered from heat shock, thus generating the cellular memory of stress [10].

In addition to prions, yeast cells contain non-heritable protein aggregates termed mnemons. While molecular foundations of prions and mnemons are similar to each other, mnemons stay in a mother cell and are not transmitted to daughters during cell division [11]. Mnemon formed by the Whi3 protein is responsible for the memory of failed mating [12]. We have previously demonstrated that a detergent-resistant aggregate of Ste18, the γ subunit of the heterotrimeric G-protein complex, which is also involved in the mating pathway, could be induced by protein overproduction and behaves as a non-heritable mnemon [13].

Heritability of yeast prions (in contrast to mnemons) is dependent on their interaction with the chaperone machinery. The concerted action of the cytosolic chaperones Hsp104, Hsp70-Ssa, and Hsp40 (Sis1 or Ydj1) results in fragmentation of amyloid fibrils into oligomers, initiating new rounds of fibril growth [14]. This process is responsible for the propagation of most yeast amyloid-based prions and their transmission in cell divisions. At least in the case of Sup35 prion ([*PSI*^+^]), Sis1 and Ssa bind to fibrils first and then recruit Hsp104, which pulls out Sup35 molecules, thus promoting fibril fragmentation [15,16]. Therefore, balance between these chaperones is crucial for the prion propagation and transmission. Both inactivation and overproduction of Hsp104 lead to the loss of [*PSI*^+^]; Hsp104 inactivation also eliminates most other yeast amyloid-based prions, although some of them are not sensitive to Hsp104 overproduction [14,17].

In addition to Ssa, yeast cells contain another cytosolic member of the Hsp70 family, Ssb, which is ribosome-associated and directly participates in co-translational folding of nascent polypeptide chains [18]. This function of Ssb requires interaction with the ribosome-associated chaperone complex (RAC), composed of Hsp40-Zuo1 and noncanonical Hsp70, Ssz1 [19,20]. The Ssb-RAC complex is also associated with the recruitment of ubiquitin ligase Ltn1, the major component of ribosome-associated protein quality control (PQC) that detects aberrations in nascent chains and leads to their ubiquitination and degradation [21]. Ssb is encoded by two nearly identical genes, *SSB1* and *SSB2* [22]. We have previously demonstrated that both spontaneous and overproduction-induced formation of the Sup35 prion, [*PSI*^+^], are increased in cells with both Ssb-coding genes deleted, *ssb1/2Δ* [23]. Accordingly, Ssb overproduction enhances [*PSI*^+^] elimination in the presence of excess Hsp104 [23] and antagonizes some variants of [*PSI*^+^] on its own [24,25]. Deletion of either *SSZ1* or *ZUO1* (or both) promotes formation of [*PSI*^+^], similar to *ssb1/2Δ* [26,27]; however, *ssz1Δ* and/or *zuo1Δ* antagonize propagation of at least some [*PSI*^+^] variants [27]. Notably, the latter effect depends on the presence of Ssb, suggesting that it occurs due to relocation of Ssb from the ribosome to cytosol in RAC-deficient strains. We have demonstrated that cytosolic Ssb interferes with binding of Ssa to the Sup35 prion aggregates, which may explain its antagonistic effect on [*PSI*^+^] propagation [27,28]. Partial relocation of Ssb from the ribosomes to cytosol was also detected in some unfavorable conditions, including heat shock, and coincided with destabilization of some [*PSI*^+^] variants [27,29]. Notably, heat-shock-induced destabilization of [*PSI*^+^] was greatly increased in the cells with RAC alterations (*zuo1Δ* and/or *ssz1Δ*) but decreased in the *ssb1/2Δ* cells, implicating Ssb relocation as a major factor modulating prion maintenance during stress [29]. Recent work by Wickner lab [30] demonstrated that the majority of [*PSI*^+^] isolates obtained in the *ssb1/2Δ* or *zuo1Δ* strains are destabilized by the reintroduction of Ssb or Zuo1, respectively. These data consistently point to the role of Ssb and RAC as an anti-prion system, antagonizing formation and propagation of the [*PSI*^+^] prion. However, the deletion of gene coding for the RAC component Ssz1 does not impact the formation of [*URE3*] prion [30], leading to the suggestion that the anti-prion effects of Ssb and RAC could be restricted to the proteins intimately associated with translational machinery, such as Sup35.

Here, we present the results of systematic analysis of the effects of Ssb on the formation and propagation of self-perpetuating aggregates by proteins that are not associated with translational machinery, namely Lsb2, Ste18 and Ure2. Our data show that Ssb is a major modulator of the formation and propagation of a variety of self-perpetuating protein aggregates in yeast.

## 2. Results

### 2.1. Effects of Ssb on the Lsb2 Aggregation and Prion Formation

**Figure 1:**
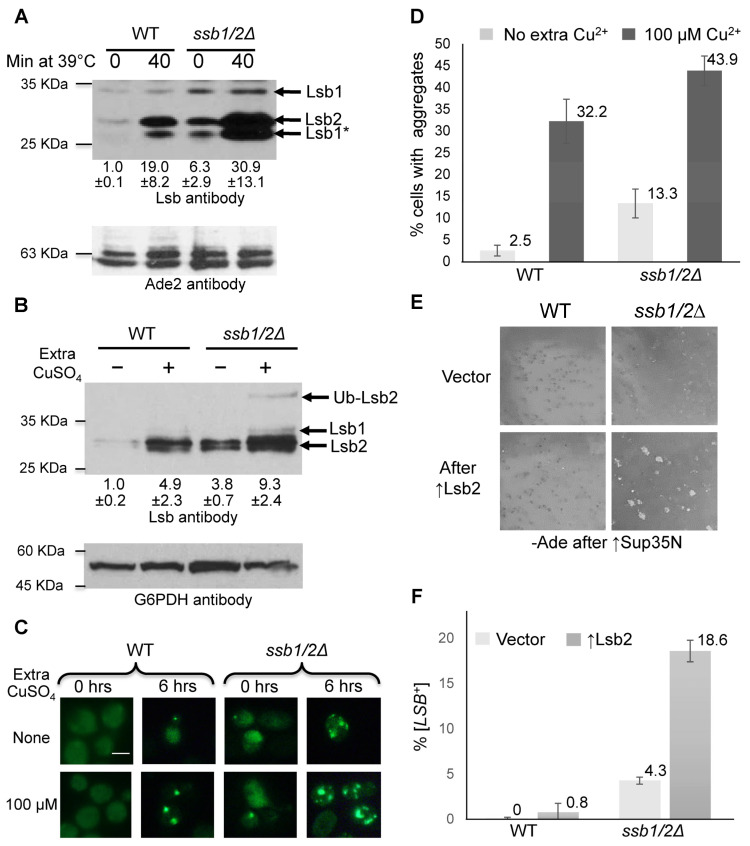
Effects of *ssb1/2Δ* deletion on Lsb2 protein levels, aggregation, and prion induction. (**A**,**B**) Comparison of the levels of Lsb2 protein in WT and *ssb1/2Δ* strains before and after 40 min heat shock at 39 °C (**A**) and with (in the presence of 150 µM CuSO_4_) or without overexpression of *LSB2* from the Cu^2+^-inducible promoter, *P_CUP1_* (**B**). Proteins were detected by SDS-PAGE and Western blotting, followed by reaction to Lsb-specific antibodies, which recognizes Lsb2, ubiquitinated Lsb2 (Ub-Lsb2, as seen in the *ssb1/2Δ* sample upon overproduction in panel (**B**), and full-length or processed (Lsb1*) isoforms of Lsb1 as indicated. In panel (**B**), Lsb1* isoform is not separable from Lsb2 but should constitute a minor fraction in the absence of heat shock. The Ade2 (**A**) or G6PDH (**B**) proteins were used as loading controls. The second band seen on the Ade2 image represents a proteolytic product, detected in most experiments. Numbers under the gel indicate the results of normalized densitometry measurements shown as a ratio to the normalized Lsb2 levels in the WT strain without heat shock (a mean of at least three repeats with standard deviation is shown in each case). Positions of the molecular weight markers are indicated on the left of each gel. (**C**,**D**) Formation of cytologically detectable aggregates of GFP-tagged Lsb2, expressed from the Cu^2+^-inducible *P_CUP1_* promoter is increased in the *ssb1/2Δ* cells. In panel (**D**), means and standard deviation are shown for three cultures in each strain/plasmid combination, after incubation in the liquid -Ura medium with or without extra Cu^2+^ added (as indicated) for 24 h at 30 °C. Scale bar corresponds to 5 µM. See Table S1 for numbers. (**E**,**F**) Induction of [*LSB*^+^] prions after transient production of HA-Lsb2 from the *P_CUP1_* promoter plasmid with or without addition of 150 μM CuSO_4_ in WT and *ssb1/2Δ* strains. Means and standard deviations are shown for at least four cultures in each strain/plasmid combination. See Table S2 for numbers.

Because Ssb is known to counteract the formation and propagation of the [*PSI*^+^] prion, we checked if it influences self-perpetuating aggregates formed by other proteins. Lsb2 is a cytoskeleton-associated protein that forms a metastable prion, [*LSB*^+^], after transient overproduction or in response to heat stress [10,31]. While the [*LSB*^+^] prion has no obvious phenotypic manifestation, it can be detected by its ability to promote [*PSI*^+^] formation in the presence of excess Sup35 or Sup35N in [*pin*^−^] cells lacking any other pre-existing prions, such as [*PIN*^+^], a prion form of the Rnq1 protein [32,33]. Aggregation of Lsb2 can also be detected biochemically and, in the case of the fluorophore-tagged Lsb2 derivative, cytologically. Lsb2 is a short-lived heat-inducible protein. We have demonstrated that levels of Lsb2 are increased in the cells lacking Ssb, both at normal and increased temperatures, when chromosomal *LSB2* is expressed from its endogenous promoter (Figure 1A) and in the absence or presence of additional Cu^2+^ when plasmid-borne *LSB2* is expressed from the copper-inducible *P_CUP1_* promoter (Figure 1B). Additionally, microscopic observations showed that a proportion of cells with cytologically detectable aggregates of GFP-tagged Lsb2 was increased in the *ssb1/2Δ* strain compared to the isogenic wild-type (WT) strain, both at low and increased levels of Cu^2+^ (Figure 1C,D and Table S1).

**Figure 2:**
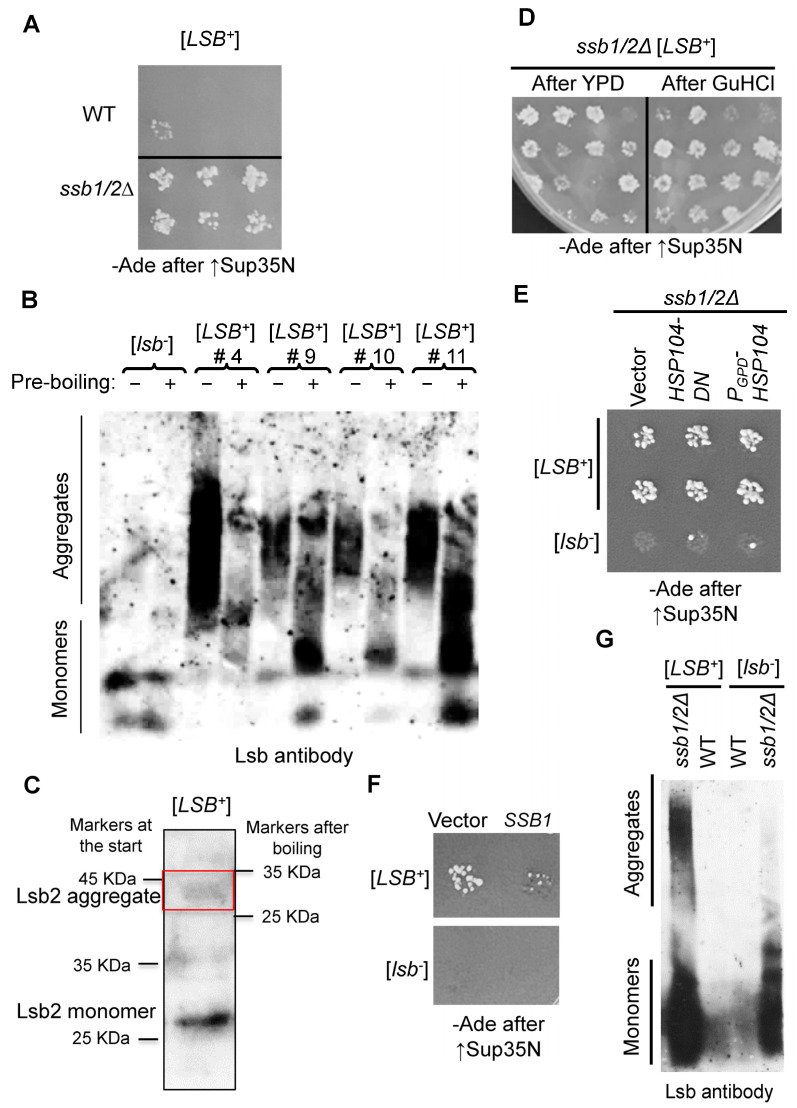
Mitotic stability and aggregate accumulation by the [*LSB*^+^] prion in the presence and absence of Ssb. (**A**) Comparison of mitotic stabilities of the [*LSB*^+^] prions obtained in WT and *ssb1/2Δ* strains, showing that most subcolonies obtained from the *ssb1/2* [*LSB*^+^] (but not from the WT [*LSB*^+^]) isolate retain their ability to promote [*PSI*^+^] formation, indicating the presence of the [*LSB*^+^] prion. For numbers, see Table S3. (**B**) Detection of the Lsb2 protein aggregates in the [*LSB*^+^] strains and their solubilization by boiling. Proteins were isolated from the *ssb1/2Δ* [*LSB*^+^] and original [*lsb*^−^] strain, bearing the *P_CUP1_-HA-LSB2* plasmid but grown without extra CuSO_4_ and heat-shocked for 40 min at 39 °C in order to maximize the Lsb2 levels without overexpressing plasmid-borne *LSB2*. Pre-boiled (+) or not pre-boiled (−) samples were fractionated on the SDD-AGE gel, followed by a reaction to the Lsb antibody. (**C**) Detection of the Lsb2 protein aggregates in the [*LSB*^+^] isolate after the loss of *P_CUP1_-HA-LSB2* plasmid by the ‘boiled gel” assay (see Section 4). Positions of molecular weight markers loaded at the start of electrophoresis (**left**) or after boiling the gel (**right**) are shown; Lsb2 monomers and aggregates are indicated; red square emphasizes the aggregate band. (**D**) Retention of the [*LSB*^+^] prion in subcolonies obtained by streaking on -Trp medium (selective for the resident *TRP1 P_GAL_-SUP35N* plasmid) after three passages on either YPD medium or YPD with 2 mM GuHCl, as indicated. Presence of [*LSB*^+^] in the subcolonies was checked by the [*PSI*^+^]-induction assay (see Section 4). The representative example is shown. See Table S4 for numbers. (**E**) Transient inactivation of Hsp104 by dominant negative *HSP104-DN* allele or overexpression of wild-type Hsp104 (both produced from the highly expressed *P_GPD_*) promoter does not affect the [*LSB*^+^] prion in the *ssb1/2Δ* background. The experiment was performed by mating assay as described in Section 4. Five [*LSB*^+^] subcolonies from each of the two independent [*LSB*^+^] isolates were tested, with similar results. A representative image with two [*LSB*^+^] subcolonies and [*lsb*^−^] control is shown. (**F**) Reintroduction of Ssb1 antagonizes the [*LSB*^+^] prion, obtained in the *ssb1/2Δ* background. The experiment was performed by mating assay as described in Section 4. Five independent [*LSB*^+^] isolates were tested, and four of them exhibited an inhibitory effect of Ssb on [*LSB*^+^]. A representative example is shown. (**G**) Reintroduction of Ssb abolishes the detergent-resistant Lsb2 aggregates. The [*LSB*^+^] and [*lsb*^−^] isolates of the *ssb1/2Δ* strain were mated with the isogenic [*psi^−^ pin*^−^] WT and *ssb1/2Δ* strains of the opposite mating type and grown in the absence of extra CuSO_4_, followed by protein isolation, fractionation on the SDD-AGE gel and reaction to the anti-Lsb antibody.

Next, we employed an assay for [*PSI*^+^] cross-seeding in order to compare frequencies of the induction of [*LSB*^+^] prions by transient overproduction of Lsb2 from the *P_CUP1_* promoter in the WT and *ssb1/2Δ* strains. Both strains were lacking any detectable pre-existing prions ([*psi^−^ pin*^−^]) and contained the plasmid with DNA fragment coding for the prion domain of the Sup35 protein (Sup35N) placed under the control of the galactose-inducible (*P_GAL_*) promoter. Lsb2 overproduction was induced by growth in the presence of increased concentrations of CuSO_4_ in the glucose medium where the *P_GAL_-SUP35N* was silent. After overproduction, cells were plated onto the solid glucose medium with a low concentration of CuSO_4_ (where both *P_CUP1_-LSB2* and *P_GAL_-SUP35N* are turned off). Grown colonies were transferred to the galactose medium where *P_GAL_-SUP35N* construct is induced, followed by velveteen-replica-plating to the glucose medium lacking the adenine (-Ade) medium for [*PSI*^+^] detection. Sup35N overproduction efficiently induces formation of [*PSI*^+^] prion only in the presence of another aggregated protein (in this case, Lsb2). [*PSI*^+^] cells could be detected by their ability to grow on -Ade medium due to the impaired termination function of the Sup35 protein in prion form, resulting in readthrough of the *ade1-14* (UGA) reporter construct [4]. Thus, growth on -Ade following the Sup35N overproduction indicated the presence of the Lsb2 prion, [*LSB*^+^], in the cells of a respective colony. Notably, formation of the phenotypically detectable [*LSB*^+^] prions after transient overproduction of Lsb2 was significantly increased in the *ssb1/2Δ* background compared to the isogenic WT strain (Figure 1E,F and Table S2). The [*LSB*^+^] isolates obtained in the WT strain were highly unstable and essentially completely lost the [*LSB*^+^] prion after colony purifying (Figure 2A and Table S3), as described previously [10]. In contrast, the [*LSB*^+^] isolates obtained in the *ssb1/2Δ* strain exhibited high mitotic stability (Figure 2A and Table S3), with most colonies retaining the [*LSB*^+^] prion even after loss of the *P_CUP1_-LSB2* plasmid. Notably, all five tested [*LSB*^+^] isolates obtained in the *ssb1/2Δ* background contained detergent-resistant Lsb2 aggregates (see examples in Figure 2B), detectable by semi-denaturing detergent agarose gel electrophoresis, SDD-AGE [34], and four of them retained detectable aggregates after the loss of the *P_CUP1_-LSB2* plasmid as shown by both SDD-AGE and “boiled gel” SDS-PAGE (see an example in Figure 2C). Lsb2-reactive material was shifted to the lower-molecular-weight fraction after pre-boiling the sample, before loading it onto the SDD-AGE gel (Figure 2B). This indicates that Lsb2 aggregates are at least partially solubilized by pre-boiling, confirming their non-covalent nature. Surprisingly, the [*LSB*^+^] isolates generated in the *ssb1/2Δ* background were not curable by growth in the presence of the anti-prion agent, GuHCl (Figure 2D and Table S4). In contrast, the [*PSI*^+^] prions formed in the *ssb1/2Δ* [*LSB*^+^] strain were curable by GuHCl as expected (Figure S1 and Table S5). GuHCl is an inhibitor of the chaperone Hsp104 [35,36], playing a crucial role in the propagation of [*PSI*^+^] and most other amyloid-based yeast prions known to date [4,14]. We have also tested the effect of Hsp104 on the [*LSB*^+^] prions, obtained in the *ssb1/2Δ* background. Neither expression of the dominant negative allele of *HSP104* (*HSP104-DN*) nor constitutive overexpression of *HSP104* from the *P_GPD_* promoter efficiently cured [*LSB*^+^] in the *ssb1/2Δ* strain (Figure 2E). This was in contrast to the Rnq1 prion, [*PIN*^+^], which was efficiently cured by *HSP104-DN* in the same experimental design (Figure S2). Thus, at least in the *ssb1/2Δ* background, the [*LSB*^+^] prions are completely or partially independent of Hsp104. Notably, mating the *ssb1/2Δ* [*LSB*^+^] strain either to the isogenic Ssb^+^ [*lsb*^−^] strain of the opposite mating type or to the isogenic *ssb1/2Δ* [*lsb*^−^] strain of the opposite mating type with the plasmid-borne *SSB1* gene (Figure 2F) led to a significant decrease in [*PSI*^+^]-inducing activity, indicating the high frequency of loss of the [*LSB*^+^] prion. Likewise, Lsb2 aggregates detectable by SDD-AGE disappeared after mating the *ssb1/2* [*LSB*^+^] strain to the WT (Ssb^+^) stain of the opposite mating type, but not after mating to the isogenic *ssb1/2Δ* strain (Figure 2G). These results show that Ssb antagonizes the propagation of [*LSB*^+^] prions generated in the absence of Ssb.

### 2.2. Effects of Ssb on the Altered Derivatives of Lsb2

**Figure 3:**
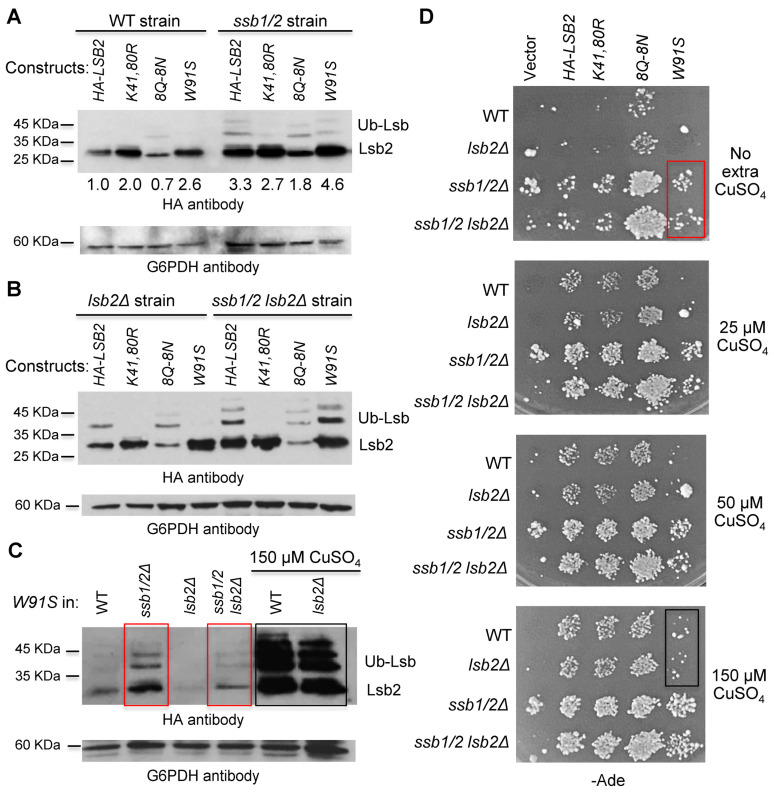
Effects of mutations on the Lsb2 protein levels and prion formation in the presence and the absence of the Ssb protein. (**A**,**B**) Levels of HA-reactive material in the Lsb^+^ (**A**) and *lsb2Δ* (**B**) strains expressing the wild-type *LSB2* gene or its mutant (*K41,80R, 8Q-8N*, and *W91S*) derivatives (as indicated) from the *P_CUP1_* promoter on a plasmid when grown with the addition of 150 µM CuSO_4_. Proteins were detected by SDS-PAGE and Western blotting, followed by a reaction to HA antibody. Positions of the molecular weight markers and of ubiquitinated Lsb2 (Ub-Lsb2) are indicated. Densitometry values calculated for normalized HA-Lsb2 and its derivatives relative to normalized wild-type HA-Lsb2 levels in the WT strain are provided for the gel shown in panel (**A**) as an example. (**C**) Comparison of the levels of WT and W91S mutant HA-Lsb2 derivatives expressed from the *P_CUP1_* promoter at low and high (after addition of 150 µM CuSO_4_) levels of Cu^2+^ both in the presence and absence of chromosomal *LSB2* and/or *SSB1/2*. In panels (**A**–**C**), the levels of G6PDH protein in the same samples are shown as a loading control. (**D**) Effects of the Lsb2 mutations on [*LSB*^+^] induction as detected in the sequential overproduction protocol (see Figure S3). Respective constructs were expressed from the *P_CUP1_* promoter in the presence of indicated amounts of CuSO_4_ in the WT and *ssb1/2Δ* strains, either containing or lacking chromosomal *LSB2* (as shown) and bearing the *P_GAL_-SUP35N* plasmid. This was followed by turning the *P_CUP1_*-mediated overexpression off and turning the *P_GAL_-SUP35N* overexpression on after velveteen-replica-plating yeast cultures to the galactose medium lacking extra CuSO_4_. Then, plates were velveteen-replica-plated to -Ade allowing detection of [*LSB*^+^] by its ability to induce [*PSI*^+^]. Squared are protein levels (**C**) and [*PSI*^+^]-induction results (**D**) for cultures expressing HA-Lsb2-W91S at high levels in the Ssb^+^ background (black squares) and cultures expressing HA-Lsb2-W91S at low levels in the *ssb1/2Δ* background (red squares).

Next, we checked if mutational alterations of Lsb2 impact the effect of Ssb on prion formation. For this purpose, we employed a series of the mutant derivatives of HA-tagged Lsb2 protein, expressed from the *P_CUP1_* promoter in WT and *ssb1/2Δ* cells, either in the presence or in the absence of the endogenous chromosomal copy of *LSB2*. Examples shown in Figure 3A,B and densitometry analysis of data indicated that levels of HA-Lsb2 induced by CuSO_4_ were increased 3.3-fold in the example shown in Figure 1A and at 1.8 -fold on average in the *ssb1/2Δ* background, compared to the Ssb^+^ cells. This increase was statistically significant (*p* < 0.01 according to sign test [37]) as it was observed in all eight repeats of the experiment. This confirms that levels of *LSB2* are elevated in the absence of Ssb even when it is expressed exclusively from a heterologous promoter. The following mutants were tested in our work: (a) double substitution K41R K80R (designated here and further as K41, 80R), knocking out major ubiquitination sites of Lsb2; (b) the 8Q-8N derivative, having the stretch of eight Q residues at positions 172–179 substituted by a stretch of eight N residues and known to increase the mitotic stability of [*LSB*^+^] prions [10,31]; and (c) the W91S substitution, disrupting the interaction of Lsb2 with actin cytoskeleton through the Las17 protein and shown by us to knock out Lsb2 aggregation and its ability to cross-seed [*PSI*^+^] [31].

In line with previous observations [31], the K41,80R derivative of HA-Lsb2 demonstrated an increase in protein levels (see examples in Figure 3A,B) in comparison to the WT protein in only five out of eight repeats when induced by CuSO_4_ from the *P_CUP1_* promoter in the Ssb^+^ strains. However, the ubiquitinated Lsb2 bands disappeared in the K41,80R mutant, confirming a defect in ubiquitination (Figure 3A,B). The Cu^2+^-induced W91S derivative of the HA-Lsb2 derivative exhibited a statistically significant increase in protein levels (by 1.7-fold on average) compared to WT HA-Lsb2 in eight out of eight repeats (*p* < 0.01) for the Ssb^+^ strain (see examples in Figure 3A,B). Neither the K41,80R nor W91S derivative showed a systematic difference in levels from the WT protein at high levels of induction in the absence of Ssb. Notably, at background levels of CuSO_4_, the W91S HA-Lsb2 derivative was accumulated at about four-fold higher levels in the *ssb1/2Δ* cells, compared to Ssb^+^ (Figure 3C). Interestingly, the Cu-induced 8Q-8N HA-Lsb2 derivative was consistently expressed at lower levels (about 0.6-fold on average) compared to the WT protein in both Ssb^+^ and *ssb1*/2Δ strains (see examples in Figure 3A,B).

Next, we tested the impact of mutational alterations on the formation of the Lsb2 prion in strains containing or lacking Ssb (Figure 3D). For this purpose, we applied the sequential induction protocol [10]. This protocol employs cultures containing both the *LSB2* constructs under the *P_CUP1_* promoter and the *SUP35N* construct under the *P_GAL_* (galactose-inducible) promoter. The *P_CUP1_* constructs are either expressed at low levels or induced at high levels on glucose medium containing low or increased concentrations of Cu^2+^, respectively. This is followed by replica-plating onto a medium containing galactose with low concentration of Cu^2+^ (Figure S3). Therefore, formation of [*PSI*^+^], used as an indicator of the presence of [*LSB*^+^], is detected under conditions of low expression of Lsb2, when [*PSI*^+^] could be induced only in the presence of a heritable [*LSB*^+^] prion. This enables us to monitor induction of heritable prions by transient overexpression of Lsb2. In line with previous observations [10,31], our data (Figure 3D) demonstrated that in the presence of Ssb, the K41,80R substitution did not significantly increase [*LSB*^+^] formation in comparison to WT Lsb2, while 8Q-8N substitution led to a significant increase (even in the absence of extra CuSO_4_), possibly due to increased mitotic stability of a prion. For both mutant derivatives, [*LSB*^+^] formation was increased further in the absence of Ssb (Figure 3D). This suggests that the increase in [*LSB*^+^] formation in the absence of Ssb is not solely due to an increase in the mitotic stability of [*LSB*^+^] because such an increase is observed even in the 8Q-8N derivative that normally produces prions with high mitotic stability.

In agreement with our previous data [31], the W91S derivative of HA-Lsb2 promoted [*LSB*^+^] formation neither in the presence nor in the absence of WT Lsb2 in the Ssb^+^ strain (Figure 3D). However, the W91S derivative of HA-Lsb2 was able to promote the formation of [*LSB*^+^] prion in the *ssb1/2Δ* background, more efficiently when endogenous Lsb2 was present (Figure 3D). Moreover, HA-Lsb2-W91S induced the formation of [*LSB*^+^] in the *ssb1/2Δ* strains even at low concentrations of Cu^2+^ (Figure 3D), despite the levels of the HA-Lsb2-W91S protein in these cells being significantly lower than in the Ssb^+^ cells grown at high concentrations of Cu^2+^ (Figure 3C) where promotion of [*LSB*^+^] formation by this protein was not detected (Figure 3D). These results confirm that the absence of Ssb indeed enhances prion-forming propensities of Lsb2-derived constructs, rather than simply operating via an increase in protein levels.

### 2.3. Prion Induction by Heat Stress in the ssb1/2Δ Background

**Figure 4:**
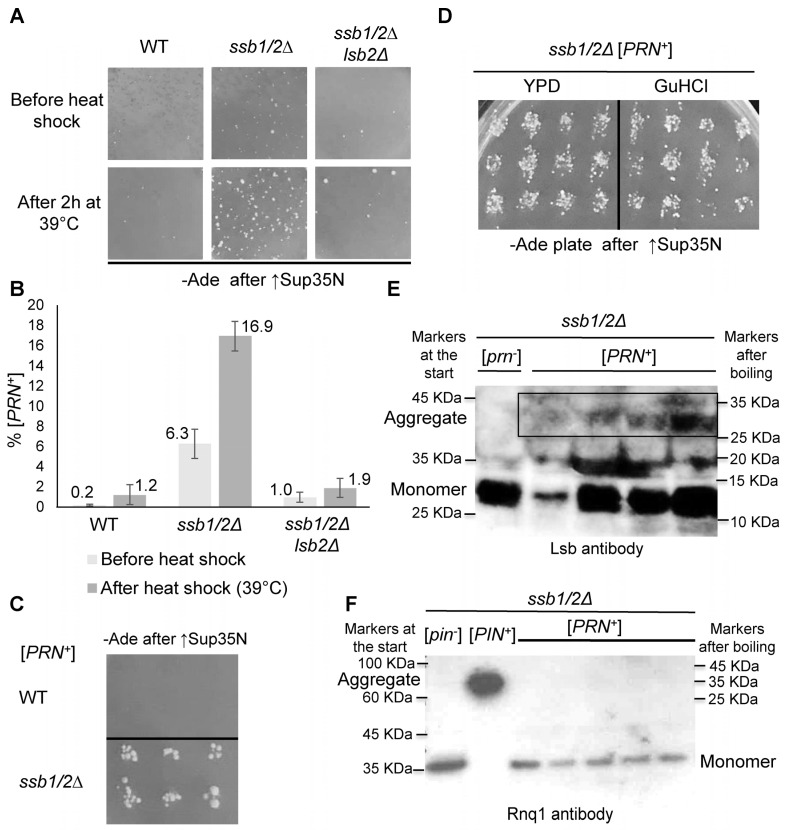
Effect of *ssb1/2Δ* on prion formation during heat shock. (**A**,**B**) Induction of derivatives with [*PSI*^+^]-inducing capacity ([*PRN*^+^]) during heat shock in the WT and *ssb1/2Δ* strains, either containing or lacking *LSB2*. Colonies obtained from heat-treated (2 h at 39 °C) and control cells are shown on -Ade medium after overexpression of *P_GAL_-SUP35N* construct are shown in panel (**A**), while frequencies of [*PRN*^+^] colonies are shown in panel (**B**); error bars indicate standard deviation. Frequency of [*PRN*^+^] colonies is increased in the *ssb1/2Δ* background, but this increase depends on *LSB2*. For numbers, see Table S6. (**C**) Mitotic stability of the [*PRN*^+^] isolates is increased in the *ssb1/2Δ* strain. Subcolonies of one typical WT [*PRN*^+^] isolate and one typical *ssb1/2Δ* [*PRN*^+^] isolate are shown as an example. For numbers, see Table S3. (**D**) [*PRN*^+^] prion is not curable by GuHCl in the *ssb1/2Δ* background. Procedure is the same as shown in Figure 2D. Subcolonies of one typical *ssb1/2Δ* [*PRN*^+^] isolate are shown as an example. For numbers, see Table S4. (**E**,**F**) Most [*PRN*^+^] prions contain aggregates of Lsb2 but not of Rnq1. Examples of the “boiled gel” analysis of the *ssb1/2Δ* [*PRN*^+^] isolates are shown with Lsb (**E**) and Rnq1 (**F**). Positions of molecular weight markers loaded at the start of electrophoresis are shown on the (**left**), and positions of molecular weight markers loaded after boiling the gel are shown on the (**right**). Positions of Lsb2 (visible aggregates are boxed) and Rnq1 monomers and aggregates are indicated.

Our previous data indicated that the formation of the [*LSB*^+^] prion is induced by heat stress [31], while *ssb1/2Δ* increases mitotic stability of the [*PSI*^+^] prion after heat shock [29]. Therefore, we checked if the generation of prions capable to cross-seed de novo formation of [*PSI*^+^] (as described for [*LSB*^+^]) after heat stress is increased in the absence of Ssb. For this purpose, the cultures of WT, *ssb1/2Δ*, and *ssb1/2Δ lsb2Δ* strains lacking known prions ([*pin*^−^]) and bearing the *TRP1 P_GAL_-SUP35N* construct were grown in complete YPD medium and incubated for 2 h at 39 °C. Samples of both heat-stressed culture and a culture before treatment were then plated onto glucose -Trp medium at 30 °C, followed by the induction of *P_GAL_-SUP35N* on galactose medium, -Trp+Gal (see Section 4). As overexpression of Sup35N promotes formation of [*PSI*^+^] only in the presence of another prion, growth on -Ade medium after galactose induction was indicative of the generation of a prion (capable of cross-seeding [*PSI*^+^] and designated [*PRN*^+^]) in the progenitor cell for a given colony. Our data confirmed that heat stress increases formation of [*PRN*^+^] prions and showed that both spontaneous and heat-induced formation of [*PRN*^+^] is significantly increased in the *ssb1/2Δ* culture compared to the WT culture (Figure 4A,B). Notably, only a slight (and in the case of heat stress, statistically insignificant) increase in [*PRN*^+^] formation was detected in the *ssb1/2Δ lsb2Δ* strain compared to the WT strain (Figure 4A and Table S6). These results indicate that *ssb1/2Δ* greatly promotes formation of new prions in yeast cells in an Lsb2-dependent manner. Increased formation of [*PRN*^+^] was especially pronounced after heat stress, when up to 17% of the *ssb1/2Δ* cells contain such a prion. About two-thirds of [*PRN*^+^] isolates obtained in the *ssb1/2Δ* background were characterized by moderate (12–28%) mitotic stability (Figure 4C and Table S3), while rare [*PRN*^+^] isolates obtained in the WT strain were completely unstable and lost [*PRN*^+^] in essentially all mitotic progeny. Similar to the [*LSB*^+^] prions induced by transient overproduction of Lsb2, the mitotic loss of [*PRN*^+^] isolates obtained in the *ssb1/2Δ* background was not increased in the presence of GuHCl (Figure 4D and Table S4). In contrast, [*PSI*^+^] prions cross-seeded by these [*PRN*^+^] isolates were curable by GuHCl in these same strains (Table S5). Seven (87%) out of eight *ssb1/2Δ* [*PRN*^+^] isolates tested contained detergent-resistant Lsb2 aggregates as shown by an example of “boiled” SDS-PAGE gel [38] (see Section 4) in Figure 4E. This confirms that in a majority of the cases, a [*PRN*^+^] prion generated during heat shock is in fact an [*LSB*^+^] prion. It should be noted that the concentration of Lsb2 aggregates in the extracts of [*PRN*^+^] cultures was usually relatively low and comparable to cells containing the proven [*LSB*^+^] prion (induced by artificial overproduction of the Lsb2 protein) after the loss of the *LSB2* plasmid (see above, Figure 2C). Therefore, it is possible that even the exceptional [*PRN*^+^] isolate not showing the Lsb2 aggregates actually contains these aggregates at even lower levels, escaping detection by the gel assay. In contrast to Lsb2, aggregates of Rnq1 (another yeast protein, known to cross-seed [*PSI*^+^] into a prion form, see [4]) were not detectable in the extracts of [*PRN*^+^] isolates by the “boiled” SDS-PAGE gel assay (Figure 4F). This confirms that the formation of [*PRN*^+^] is typically not due to generation of a prion form of Rnq1.

### 2.4. Effects of Ssb on Ste18 Aggregation

**Figure 5:**
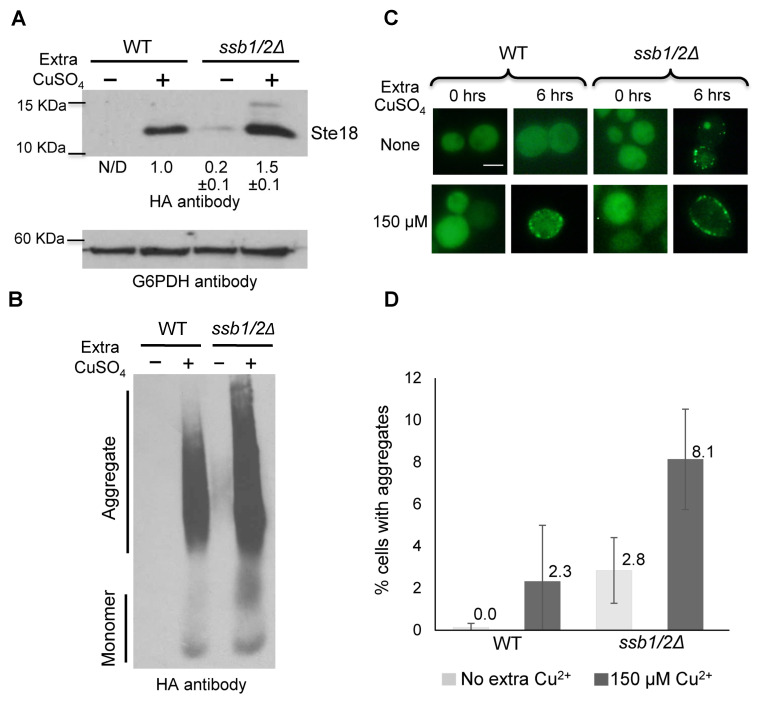
Effects of Ssb on levels and aggregation of the Ste18 protein. (**A**) Levels of HA-tagged Ste18 expressed from the construct under the *P_CUP1_* promoter. Proteins were isolated from cultures grown in the plasmid-selective medium for 48 h without (−) or with (+) 150 μM CuSO_4_ added and analyzed by SDS-PAGE and Western blotting, followed by reaction to HA antibodies. Positions of molecular weight markers are indicated. Levels of G6PDH protein in the same samples are shown as a loading control. Ratios of Ste18 determined by densitometry and normalized by G6DPH are indicated, relative to the level of Ste18 in the WT strain with CuSO_4_. Numbers correspond to a mean of three replicates with standard deviations. Ste18 was not detectable (N/D) in this experiment in the WT strain grown without the addition of CuSO_4_ (**B**) Detection of detergent-resistant aggregates of HA-Ste18 in the WT and *ssb1/2Δ* samples (obtained as shown in panel (**A**)) by SDD-AGE. (**C**,**D**) Detection of GFP-Ste18 aggregates by fluorescence microscopy. Cells from cultures containing the plasmid with *P_CUP1_-GFP-STE18* construct and grown with or without addition of 150 μM CuSO_4_ for indicated periods of time are shown in panel (**C**). Scale bar corresponds to 5 µM. Average frequencies (a mean of three independent replicates with a standard deviation) of cells with aggregates in cultures containing the *P_CUP1_-GFP-STE18* plasmid and grown for 6 h either with or without addition of 150 μM CuSO_4_ are shown in panel (**D**). For numbers, see Table S7.

The yeast Ste18 protein is a γ-subunit of G-protein, which is involved in the pheromone signaling pathway and is shown by us [13] to form a non-heritable amyloid-like aggregate with properties of yeast mnemon upon overproduction. Similar to Lsb2, aggregated Ste18 can cross-seed Sup35 into a prion form, [*PSI*^+^]. We have investigated the effect of *ssb1/2Δ* on Ste18 aggregation and on [*PSI*^+^] cross-seeding by Ste18. Ste18, produced from the construct under the *P_CUP1_* promoter, was essentially undetectable at low levels of Cu^2+^ in the WT strain; however, it was induced by an increased concentration of Cu^2+^. Notably, levels of Ste18 were increased in the *ssb1/2Δ* strain, both at low (so that it becomes detectable) and high (at about 1.5-fold) concentrations of Cu^2+^ (Figure 5A). As described previously [22], Ste18 overproduction at high levels of Cu^2+^ resulted in the accumulation of detergent-resistant aggregates, which could be visualized on an SDD-AGE gel and were more abundant in the *ssb1/2Δ* background (Figure 5B). Aggregates of the GFP-tagged Ste18 construct could also be detected by fluorescence microscopy, and the accumulation of cytologically detectable aggregates was increased in the *ssb1/2Δ* strain compared to the isogenic WT strain (Figure 5C,D and Table S7).

**Figure 6:**
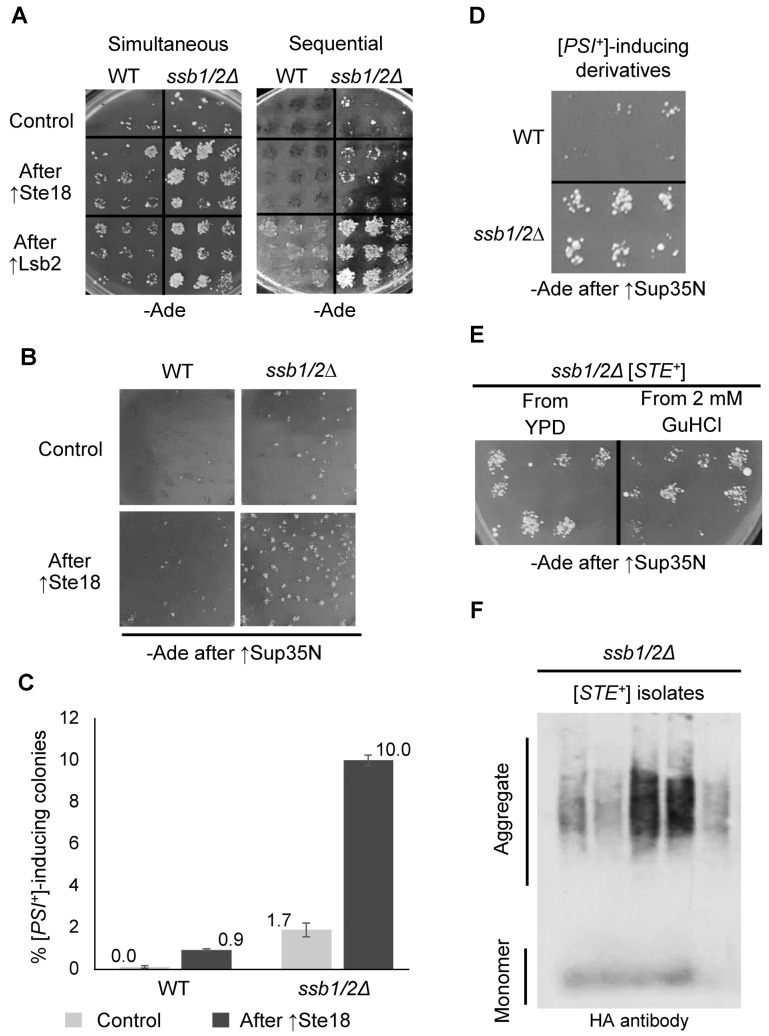
Effect of Ssb on the formation and heritability of the [*STE*^+^] derivatives. (**A**) Formation of the [*STE*^+^] derivatives (detected by the promotion of [*PSI*^+^] induction) after transient overexpression of the *P_CUP1_-HA-STE18* construct in accordance with either simultaneous (**left**) or sequential (**right**) induction protocol (see Figure S3). In the simultaneous induction protocol, both Ste18 and Sup35N are overproduced together, while in the sequential induction protocol, Ste18 is overproduced first, followed by overproduction of Sup35N under conditions when Ste18 overproduction is turned off. Ste18 overproduction promotes [*PSI*^+^] formation only in the simultaneous protocol in the WT strain but in both simultaneous and sequential induction protocols in the *ssb1/2Δ* strain, indicating that Ste18 aggregates become partly heritable in the absence of Ssb. Empty vector is shown as a negative control, while vector overproducing Lsb2, which promotes [*PSI*^+^] formation in both simultaneous and sequential induction assays in both WT and *ssb1/2Δ* strains, is shown as a positive reference. (**B**,**C**) Induction of [*STE*^+^] colonies (detected by the ability to promote [*PSI*^+^] formation) by transient overexpression of the *P_CUP_-HA-STE18* construct after the addition of 150 μM CuSO_4_ to the growth medium in the WT and *ssb1/2Δ* strains. Empty vector was used as a control. Cultures were grown for 24 h in the presence of an inducer. Percentages in panel (**C**) represent a mean of at least three independent cultures for each strain/plasmid combination, with bars showing standard deviation. For numbers, see Table S8. (**D**) The [*PSI*^+^]-inducibility phenotype of colonies shown in panel (**B**) is mitotically heritable in the *ssb1/2Δ* (but not in the WT) background. Subcolonies of one typical colony, obtained on the medium selective for the plasmid (but without Cu^2+^ induction), are shown for each WT and *ssb1/2Δ* strain. Similar results were obtained without selection for the plasmid. For numbers, see Table S9. (**E**) [*STE*^+^] prion is not curable by GuHCl in the *ssb1/2Δ* background. The experiment was performed in the same way as shown in Figure 2D and Figure 4D. A typical example is shown. For numbers, see Table S4. (**F**) The [*STE*^+^] derivatives obtained as shown in panel (**B**) contain detergent-resistant aggregates of the Ste18 protein. Proteins, isolated from [*STE*^+^] derivatives of the *ssb1/2Δ* strain, containing the *P_CUP1_-HA-STE18* plasmid and grown in the absence of extra CuSO_4_, were run on the SDD-AGE gel with sodium N-lauroylsarcosine, followed by a transfer to the nitrocellulose membrane and a reaction to the HA antibody.

Two protocols were employed for the detection of Ste18-dependent cross-seeding of [*PSI*^+^] prion (Figure S3), namely simultaneous overexpression when both Ste18 and Sup35N were co-overexpressed in the same cell and sequential overexpression when Ste18 was overexpressed first, followed by the induction of Sup35N after overexpression of Ste18 was turned off. The latter protocol was similar to one employed previously for Lsb2 and its mutant derivatives (Figure S3 and Figure 3C), and Lsb2 was used as a positive control in both protocols. In the WT strain, Lsb2 promoted [*PSI*^+^] induction after both simultaneous and sequential overexpression, while Ste18 cross-seeded [*PSI*^+^] only in the simultaneous and not the sequential protocol (Figure 6A). This result agreed with our previous data [13] and was expected as aggregates of Ste18 are entirely non-heritable in the WT strain in contrast to aggregates of Lsb2 which are heritable. However, we found that overexpressed Ste18 is able to promote [*PSI*^+^] formation both after simultaneous and after sequential overproduction in the *ssb1/2* background (Figure 6A). This indicates that Ste18 aggregates formed after Ste18 overproduction become heritable in the absence of Ssb.

Indeed, up to 10% of colonies obtained after transient overproduction of Ste18 in the *ssb1/2Δ* strain were capable of [*PSI*^+^] induction compared to less than 1% of such colonies in the WT strain (Figure 6B,C and Table S8). Moreover, most of the [*PSI*^+^]-inducing derivatives obtained in the WT background lost the ability to induce [*PSI*^+^] after colony purification, while most [*PSI*^+^]-inducing derivatives obtained in the *ssb1/2Δ* strain (and designated [*STE*^+^]) were able to transmit the [*PSI*^+^]-inducing ability to a majority of the mitotic progeny (Figure 6D and Table S9). Notably, the *ssb1/2Δ* [*STE*^+^] isolates exhibited high retention of the [*PSI*^+^]-inducing phenotype even after the loss of the *STE18* plasmid, indicating that the [*STE*^+^] prion can be maintained by the endogenous Ste18 protein (Table S9). However, mitotic stability of [*STE*^+^] was severely impaired after storage at −80 °C with subsequent recovery (Table S9). Similar to [*LSB*^+^], [*STE*^+^] isolates obtained in the *ssb1/2Δ* strain were not curable by GuHCl (Figure 6E and Table S4), while [*PSI*^+^] isolates cross-seeded by [*STE*^+^] were curable (Figure S1 and Table S5). SDD-AGE analysis confirmed that [*STE*^+^] isolates bearing the *P_CUP1_-HA-STE18* construct and grown in the absence of extra Cu^2+^ contain a fraction of the Ste18 protein in the form of detergent-resistant aggregates (Figure 6F). Therefore, Ste18 aggregates behaved as heritable prions in the *ssb1/2Δ* background.

### 2.5. Effects of Ssb and Zuo1 on the [URE3] Prion

**Figure 7:**
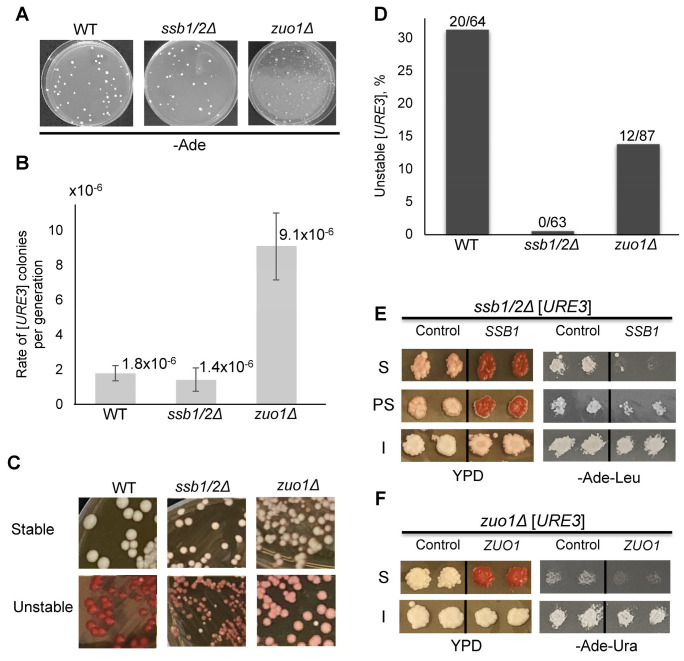
Effects of Ssb and Zuo1 proteins on the [*URE3*] prion. (**A**) Examples of -Ade plates with approximately equal amounts of cells plated, showing spontaneous formation of presumable [*URE3*] (Ade^+^) colonies in the isogenic WT, *ssb1/2Δ* and *zuo1Δ* strains. (**B**) Rates of [*URE3*] formation in the isogenic WT, *ssb1/2Δ*, and *zuo1Δ* strains, determined from fluctuation test. Error bars represent the 95% confidence limits. For frequencies and numbers, see Table S10. (**C**) Examples of mitotic stabilities of [*URE3*] prions arisen spontaneously in isogenic WT, *ssb1/2Δ*, and *zuo1Δ* strains. In each case, an Ade^+^ colony was streaked out for single subcolonies on YPD medium. Prion retention and loss were detected by the whitish and reddish color of subcolonies, respectively. (**D**) Distribution of unstable derivatives among the [*URE3*] isolates, arisen spontaneously in the WT, *ssb1/2Δ*, and *zuo1Δ* strains. For more detailed information, see Table S11. (**E**) Inhibition of some [*URE3*] prions, obtained in the *ssb1/2Δ* background, by reintroduction of the plasmid bearing the *GFP-SSB1* construct (*SSB1*) under the control of the strong constitutive glyceraldehyde-3-phosphate dehydrogenase promoter (*P_GDP_*). The transformants were patched on the plasmid-selective medium (-Leu) and velveteen-replica-plated to YPD medium (for the color assay) and to -Ade-Leu medium (for the growth assay). Empty vector was used as a control. Similar results were obtained after reintroduction of the plasmid with the *SSB2* gene under its endogenous promoter. For more detailed information, see Table S13. (**F**) Inhibition of some [*URE3*] prions, obtained in the *zuo1Δ* background, by reintroduction of the plasmid bearing the *ZUO1* gene under the control of the strong constitutive EF1α (*P_TEF1_*) promoter. The transformants were patched on the plasmid-selective medium (-Ura) and velveteen-replica-plated to YPD medium (for the color assay) and to -Ade-Ura medium (for the growth assay). For more detailed information, see Tables S14 and S15. Designations in panels (**E**,**F**) are as follows: S: sensitive; PS: partially sensitive; I: insensitive to reintroduction of Ssb (**E**) or Zuo1 (**F**).

Previous data indicated that both deletion of genes coding for Hsp70-Ssb (*ssb1/2Δ*) and deletion of either RAC component (*ssz1Δ* or *zuo1Δ*) increase the formation of the [*PSI*^+^] prion [23,26,27,30], while our new data (see above) show that *ssb1/2Δ* also promotes the formation and heritability of the aggregated forms of the Lsb2 and Ste18 proteins. However, it has been reported previously that *ssz1Δ* does not influence the formation of the prion form of the yeast Ure2 protein, [*URE3*] [30]. Therefore, we checked if *ssb1/2Δ* or *zuo1Δ* influence [*URE3*] formation. For this purpose, respective deletions were introduced into the yeast strain, bearing the reporter construct *DAL5::ADE2*, which yields the detection of the [*URE3*] prion by growth on -Ade medium and a lighter (white or pink versus red) color on complete YPD medium [39]. Fluctuation test data indicated that *ssb1/2Δ* did not have an influence, while *zuo1Δ* significantly increased both the frequency (Figure 7A and Table S10) and rate (by five-fold, Figure 7B and Table S10) of the formation of Ade^+^ cells compared to the isogenic WT strain. Notably, WTand *zuo1Δ* strains produced higher proportion of Ade^+^ colonies exhibiting high mitotic loss of Ade^+^ phenotype, compared to the *ssb1/2Δ* strain (Figure 7C,D and Table S11). A majority of Ade^+^ colonies with high mitotic stability were curable by growth in the presence of GuHCl in all strains (Table S12). This data confirms that most Ade^+^ isolates represent non-Mendelian elements (presumably [*URE3*] prions). Overall, our results show that *zuo1Δ* increases spontaneous formation of [*URE3*], while *ssb1/2Δ* does not show a significant effect on [*URE3*] formation.

Next, we checked if the presence of Ssb influences the inheritance of [*URE3*] prions obtained in the *ssb1/2Δ* background. Such an effect was suggested by the observation that the *ssb1/2Δ* strain produced a lower proportion of highly unstable spontaneous [*URE3*] isolates compared to the WT and *zuo1Δ* strains (Figure 7D and Table S11). To investigate the effect of Ssb on [*URE3*] stability further, GuHCl-curable [*URE3*] isolates obtained in the *ssb1/2Δ* strain were transformed with a plasmid, expressing either *SSB1* from a strong constitutive *P_GP_*_D_ promoter or *SSB2* from its endogenous promoter. Typically, the [*URE3*] prion was either completely lost (two out of nine isolates tested), or completely or partially inhibited and mitotically destabilized (remaining seven isolates) after reintroduction of *SSB1* (see examples in Figure 7E and data in Table S13). Reintroduction of *SSB2* completely or partially inhibited and mitotically destabilized [*URE3*] in five out of six isolates tested (Table S13). Notably, introduction of the same *SSB1* or *SSB2* plasmids did not have any significant impact on the [*URE3*] isolates obtained in the WT strain, except somewhat increasing mitotic stability in one case (Table S13). This confirms that the inhibitory effect on [*URE3*] was primarily due to reintroduction of Ssb rather than its potential overexpression from a plasmid-based gene. Overall, these data show that while Ssb does not influence spontaneous formation of [*URE3*], it does significantly impair phenotypic expression and/or propagation of most [*URE3*] isolates.

Likewise, we checked if reintroduction of *ZUO1* influences the phenotypic manifestation of Ade^+^ isolates obtained in the *zuo1Δ* background (Figure 7F and Table S14). In two out of four *zuo1Δ* Ade^+^ isolates tested, growth on -Ade was completely inhibited, and color became more reddish in the presence of *ZUO1*, expressed from the strong constitutive *P_TEF1_* promoter. Analysis of individual colonies obtained from these isolates confirmed that the [*URE3*] prion was lost after introduction of the *P_TEF1_-ZUO1* construct. No such destabilization of [*URE3*] occurred among six isolates obtained in the WT strain. Overall, our data show that at least some [*URE3*] prions obtained in the absence of Zuo1 are sensitive to the reintroduction of Zuo1, confirming the impact of an RAC component on the [*URE3*] prion.

### 2.6. Effects of Ssb on Detection and Mitotic Stability of [PSI^+^]

**Figure 8:**
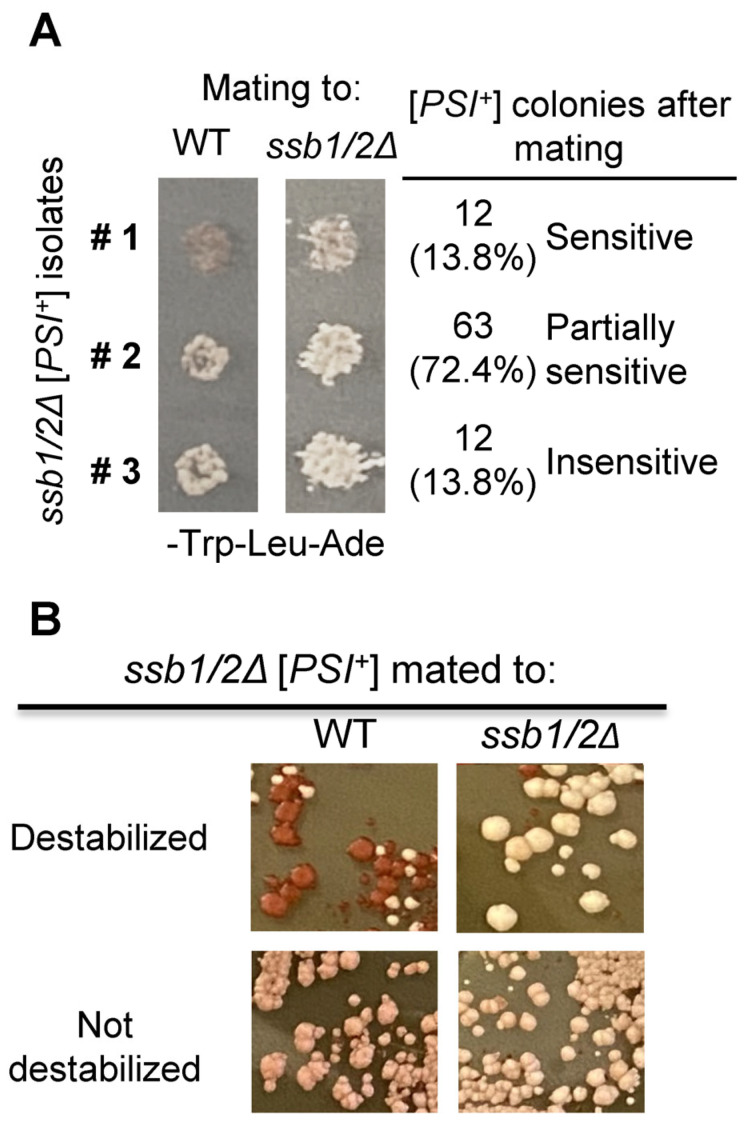
Effect of the reintroduction of Ssb on [*PSI*^+^] isolates obtained in the *ssb1/2Δ* background. (**A**) Independent *ssb1/2Δ* [*PSI*^+^] derivatives with *TRP1* plasmid were mated to isogenic WT and *ssb1/2Δ* strains of the opposite mating type (*MATα*) carrying a plasmid with the complementary (*LEU2*) marker. Resulting diploids were selected on -Trp-Leu medium and velveteen-replica-plated to the -Trp-Leu-Ade to detect the presence and stringency of [*PSI*^+^]. (**B**) Mitotic stability of [*PSI*^+^] prion in diploids obtained as described above for panel (**A**). Diploids were streaked on appropriate selective media (-Trp-Ura) and velveteen-replica-plated to YPD for the color assay. Examples are shown; see text for numbers.

While the effect of Ssb on the [*PSI*^+^] prion is well established, some discrepancies remain. For example, our previous data indicated that most of the *ssb1/2Δ*-derived [*PSI*^+^] isolates remain detectable after transformation with the *SSB1*-containing plasmid [23]. However, Son and Wickner reported that a significant fraction of [*PSI*^+^] isolates obtained in the *ssb1/2Δ* strain are not detectable after mating to the strain bearing the wild-type *SSB1* and *SSB2* alleles [30]. In order to recheck the effect of Ssb reintroduction on [*PSI*^+^] stability using the approach similar to that of Son and Wickner, we mated a sample of [*PSI*^+^] isolates, induced by transient overproduction of Sup35N in the [*psi^−^ PIN*^+^] *ssb1/2Δ* strain, to the [*pin^−^ psi*^−^] WT and *ssb1/2Δ* strains of the opposite mating type (*MAT*α). About 14% of the [*PSI*^+^] isolates lost suppression of *ade1-14* after mating, while another 72% exhibited lower suppression after mating to the WT strain compared to mating to *ssb1/2Δ* (Figure 8A). Importantly, 5 (about 40%) out of 12 tested [*PSI*^+^] isolates retaining prion exhibited decreased mitotic stability of a prion after reintroduction of Ssb (as judged from the increased appearance of red [*psi*^−^] and mosaic mixed [*PSI*^+^]/[*psi*^−^] subcolonies in their mitotic progeny) compared to mating to the *ssb1/2Δ* partner (Figure 8B). Taken together, our data demonstrate that while reintroduction of Ssb indeed decreases phenotypic manifestation of most and mitotic stability of some [*PSI*^+^] isolates obtained in the *ssb1/2Δ* background, the majority (about 86%) of these [*PSI*^+^] isolates remain detectable in the presence of Ssb, at least in the yeast strain used in our work.

## 3. Discussion

### 3.1. Comparison of the Effects of Ribosome-Associated Chaperones on [PSI^+^] and Other Prions

Previous reports by us [23,27,28,29,40] and others [24,25,26,30] indicated that alterations of the Ssb/RAC complex influence the Sup35 prion, [*PSI*^+^]. However, the deletion of at least one gene coding for an RAC component, *SSZ1*, did not show an effect on another prion, [*URE3*] [30]. This led to the suggestion that the effects of ribosome-associated chaperones are specific to the prion form of Sup35, which is a translation factor, working in association with the ribosome. However, our new data show that the lack of Ssb influences heritable aggregation of several yeast proteins, namely Lsb2 (cytoskeleton-associated protein), Ste18 (Gγ subunit in the G-coupled receptor associate signaling pathway) and Ure2 (a regulator in nitrogen metabolism). These results clearly demonstrate that Hsp70-Ssb is a general modulator of heritable aggregation of a variety of proteins in the yeast cell, including those not related to the translational apparatus.

Patterns and specific features of the Ssb effects could vary depending on the target protein. In the case of Sup35, both an increase in de novo prion formation [23] and a promotion of prion propagation after stress [29] or in normal conditions [30] in the absence of Ssb have previously been reported. Son and Wickner [30] observed that most [*PSI*^+^] prions obtained in the absence of Ssb are lost after reintroduction of Ssb by mating, suggesting that the impact of Ssb on detectable de novo [*PSI*^+^] formation could be, in significant part, due to the inability to detect Ssb-sensitive prions in the WT strain. However, our experiments using the same approach as in the Son and Wickner paper indicate that while reintroduction of Ssb indeed partially inhibits or destabilizes a majority of [*PSI*^+^] isolates generated in the *ssb1/2Δ* background, most of them remain phenotypically detectable in the Ssb^+^ diploid (Figure 8). This confirms our previous results obtained after reintroduction of *SSB1* on a plasmid [23] and supports our previous conclusion that Ssb has a dual effect on Sup35 prions, inhibiting both de novo prion formation and propagation of a pre-existing prion [27,28]. It should be noted that despite numerical differences from our results, which are likely explained by different genotypic backgrounds of yeast strains and/or using different inducer constructs, Son and Wickner calculations also agree with a notion of such a dual effect [30]. As argued in our previous work [27,28], it is likely that the inhibitory effect of Ssb on de novo prion formation is due to the promotion of the proper protein folding into non-prion conformation by Ssb, while the inhibition of prion propagation could be explained by a competition between cytosolic Hsp70-Ssb and Hsp70-Ssa (involved in prion propagation) for binding to Sup35 prion aggregates.

### 3.2. Formation and Propagation of the [LSB^+^] Prion in the Absence of Ssb

In addition to the “classic” prions of [*PSI*^+^] and [*URE3*], lack of Ssb increases de novo formation and promotes mitotic transmission of a typically metastable prion, formed by the cytoskeleton-associated protein Lsb2 (Figure 1 and Figure 2). While an effect of *ssb1/2Δ* on mitotic stability of [*LSB*^+^] prion is dramatic, it is not likely that an increased observable formation of [*LSB*^+^] is solely due to improved mitotic transmission because prion formation by the 8Q-8N mutant derivative of Lsb2, typically producing prions with high mitotic stability [10], is also increased in the *ssb1/2Δ* background (Figure 3C). This shows that, such as in the case of [*PSI*^+^], Ssb has a dual effect on both [*LSB*^+^] prion formation and propagation.

Lsb2 is a short-lived stress-inducible protein, degraded via the UPS [31]. Levels of Lsb2 are increased in the *ssb1/2Δ* strain compared to the WT strain. This increase is not solely due to the effect of *ssb1/2Δ* on *LSB2* transcription from its endogenous promoter as it is observed with both endogenous (Figure 1A) and artificial (*P_CUP1_*, Figure 1B and Figure 3A–C) promoters. It is possible that the increase in Lsb2 levels in the absence of Ssb is at least in part due to the impact of *ssb1/2Δ* on ubiquitination-dependent degradation of Lsb2; however, this question remains open as the impact of ubiquitination site knockout on Lsb2 levels is slight and not always reproducible (see Figure 3A,B). While an increase in Lsb2 levels could possibly contribute to an increase in prion formation and mitotic stability in the absence of Ssb, some results cannot be solely explained by this mechanism. Specifically, the mutant Lsb2-W91S derivative, which is not capable of binding actin cytoskeleton via Las17 and cannot produce a prion in the WT strain, acquires the prion properties in the *ssb1/2Δ* background and becomes capable of inducing [*LSB*^+^] formation even at low protein levels, even though it cannot do so at much higher levels in the Ssb^+^ strain (Figure 3C,D).

Notably, the [*LSB*^+^] prions, obtained and propagated in the *ssb1/2Δ* background, are typically curable neither by GuHCl (Figure 2C and Table S4), which is known to inhibit Hsp104 [35,36], nor by transient inactivation or overproduction of Hsp104 (Figure 2E). These data show that propagation of the [*LSB*^+^] prion in the *ssb1/2Δ* background does not require Hsp104 activity at the level required for propagation of other yeast prions, such as [*PSI*^+^] or [*PIN*^+^]. Further studies are needed to determine whether the absence of Ssb alters interactions between Hsp104 and [*LSB*^+^] aggregates or a different kind of [*LSB*^+^] prion variants is preferentially produced in the *ssb1/2Δ* background compared to the Ssb^+^ background. It should be noted that while previously we observed an increased loss of [*LSB*^+^] in the presence of GuHCl in the WT strain [10], the interpretation of this result is somewhat ambiguous, because mitotic stability of [*LSB*^+^] in the WT cells is very low even in the absence of GuHCl so the systematic characterization of a variety of [*LSB*^+^] isolates in regard to GuHCl-mediated curing was difficult. Therefore, it cannot be excluded that a significant fraction of [*LSB*^+^] isolates are not curable by GuHCl independently of the presence or absence of Ssb. Examples of yeast prions that are not dependent of Hsp104 are reported in the literature [41,42,43,44], although most of such prions were shown to be of a non-amyloid nature. While Lsb2 aggregates definitely possess some features of amyloids, such as resistance to detergents (see Figure 2B,D and Figure 4E), it is possible that they are different from “typical” amyloids and that Hsp104 is not crucial for their fragmentation.

### 3.3. Role of Ssb in Cellular Memory

Previously, we reported that [*PSI*^+^]-nucleating prions are induced in an Lsb2-dependent manner by heat stress, thus implicating metastable Lsb2 aggregates as carriers of cellular memory of stress [10,45]. Notably, the lack of Ssb significantly increases accumulation of such prions, initially designated as [*PRN*^+^] (Figure 4A,B and Table S6). We have demonstrated that in majority of the cases, the [*PRN*^+^] isolates indeed contain an aggregated form of Lsb2, implicating them as variants of [*LSB*^+^] (Figure 4E). Even a non-stressed exponentially growing *ssb1/2Δ* culture accumulated about 6% of [*PRN*^+^] cells; after heat stress, their frequency was increased to 17% (Figure 4B). This result suggests that Lsb2 is apparently a major yeast protein forming heritable aggregates in response to heat stress, while Ssb is a major chaperone downregulating the formation and propagation of these aggregates. The biological meaning of this regulation could be explained by the potentially adaptive effect of Lsb2 prion during stress as proposed in [45], coupled with its potentially detrimental impact on non-stressed cells. Thus, Ssb could be responsible for keeping the fraction of cells with stress-induced prions low, diminishing prion interference with the efficient recovery and proliferation of the majority of the culture after stress.

While mitotic stability of stress-induced [*LSB*^+^] isolates increased in the *ssb1/2Δ* strain compared to the rare and highly unstable isolates detected in the WT strain (Figure 4A and Table S3), most heat-induced *ssb1/2Δ* [*LSB*^+^] isolates were characterized by lower mitotic stability compared to most *ssb1/2Δ* [*LSB*^+^] isolates generated after artificial overproduction of Lsb2 (Table S3). This shows that either heat stress preferentially produces different variants of the [*LSB*^+^] prion compared to the prions induced by artificial Lsb2 overproduction, or simply the initially generated fraction of the aggregated Lsb2 protein is smaller after heat shock compared to plasmid-mediated overproduction of Lsb2. In the latter scenario, it is more difficult for the aggregated isoform to efficiently overtake the whole protein pool in the cells, which explains its higher loss in subsequent cell divisions.

Another yeast protein, a Gγ-subunit (Ste18), forms non-heritable detergent-resistant aggregates upon overproduction in the WT strain [13]. These aggregates resemble “mnemons” previously described for another yeast protein in the pheromone signaling pathway, Whi3 [12]. Notably, Ste18 aggregates are increased in abundance and become partly heritable in the *ssb1/2Δ* strain (Figure 5 and Figure 6), indicating that the Ste18 mnemon is converted into a heritable prion in the absence of Ssb. Such as in the case of Lsb2, Ste18 is an unstable protein degraded via the ubiquitin–proteasome system [13], and its levels are somewhat increased in the absence of Ssb (Figure 5A). However, it does not seem likely that such a relatively modest increase in levels (at about 1.5-fold at high levels of Ste18 production, Figure 5A) is sufficient to explain the promotion of both formation and heritability of Ste18 aggregates.

### 3.4. Modulation of the [URE3] Prion by Ribosome-Associated Chaperones

In the case of the [*URE3*] prion, no effect of *ssb1/2Δ* on the rate of spontaneous prion formation was detected (Figure 7 and Table S10). However, a larger proportion of mitotically unstable [*URE3*] variants were recovered in the WT background compared to the *ssb1/2Δ* strain (Figure 7C,D, and Table S11). Moreover, reintroduction of the *SSB1* or *SSB2* gene on a plasmid led to the partial or complete inhibition of the phenotypic manifestation of the vast majority of [*URE3*] isolates, generated in the *ssb1/2Δ* background (Figure 7F and Table S13). As the [*URE3*] detection assay is not based on translational readthrough, this effect could not be due to the previously described effect of *ssb1/2Δ* on nonsense suppression [23,30], which partially contributes to the inhibition of [*PSI*^+^] by Ssb. More likely, inhibition of [*URE3*] occurred due to destabilization of some prion isolates in the presence of Ssb as shown in Table S14. The observation that partial inhibition of prion manifestation and mitotic transmission by Ssb does not result in significant differences in the frequencies and rates of detectable [*URE3*] formation between the WT and *ssb1/2Δ* strains (Figure 7B and Figure S4 and Table S10) provides an additional argument in favor of the notion that the effect of Ssb on the formation of other heritable aggregates ([*PSI*^+^], [*LSB*^+^] and [*STE*^+^]) is not a simple consequence of the effects of Ssb on [*PSI*^+^] manifestation and mitotic stability.

In contrast to *ssb1/2Δ*, deletion of the gene coding for the RAC component Zuo1 (an Hsp40 cochaperone of Hsp70-Ssb) led to a significant (five-fold) increase in the rate of Ade^+^ colonies detected by the [*URE3*] reporter system (Figure 7A,B and Table S10). Most of the Ade^+^ colonies were either mitotically unstable (Figure 7C,D and Table S11) or GuHCl-curable (Figure 7E and Table S12), or both, implicating them as [*URE3*] prions. This shows that *zuo1Δ* increases spontaneous formation of the [*URE3*] prion, similar to the previously described effect of *zuo1Δ* on the formation of [*PSI*^+^] prion. It should be noted that according to our previous data [27], *zuo1Δ* decreases the mitotic stability of some [*PSI*^+^] isolates in an Ssb-dependent manner. We suggest that this occurred due to the relocation of Ssb from the ribosome to cytosol in *zuo1Δ* cells, as shown previously [46] and confirmed in our studies [27]. Cytosolic Ssb competes with Ssa involved in [*PSI*^+^] propagation [27,28]. It is likely that a similar scenario could work in the case of [*URE3*]. Notably, phenotypic manifestation and/or mitotic transmission of some [*URE3*] isolates obtained in the *zuo1Δ* background were inhibited by reintroduction of the *ZUO1* gene (Figure 7G). [*PSI*^+^] isolates with such a sensitivity to RAC components have also been described [30,47]. One possible explanation in the case of [*URE3*] is that an increased level of at least one member of the Ssa family is known to destabilize the [*URE3*] prion [48], while increased accumulation of Ssb to the cytosol in the absence of *ZUO1* could partly compensate for this effect by antagonizing Ssa.

### 3.5. Biological Relevance and Future Perspectives

Our data implicate the ribosome-associated chaperoning machinery as a universal modulator of heritable protein aggregation in the yeast cell, as components of this machinery impact formation and propagation of prions formed by various proteins. Ssb consistently counteracts heritable protein aggregation, even though specifics of its effect on various aggregating proteins vary. This is consistent with the role of Ssb as one of the perpetuators of an anti-prion defense, as discussed previously [30]. The anti-prion activity of Ssb may employ various mutually non-exclusive mechanisms, including promoting the non-prion folding of nascent polypeptides, antagonizing accumulation of potentially aggregating proteins, and inhibiting the activity of the cytosolic chaperoning machinery that is involved in the fragmentation and propagation of prion aggregates. Our previous data showing the modulation of the intracellular localization and prion-related activities of Ssb by environmental and physiological conditions [27,28,29] indicate that an anti-prion effect of Ssb is physiologically relevant and may provide a mechanism for the cross-talk between the protein biosynthesis machinery and self-perpetuating protein aggregation. This is also in line with the previously important increase in prion formation in yeast cells lacking multiple anti-prion proteins [47], and this is further confirmed by our new results showing that Ssb influences aggregates involved in cellular memory. Overall, the physiological impact of Ssb-dependent modulation of heritable aggregation and cellular memory could be quite dramatic as signified by the accumulation of a high proportion (up to 17%) of cells bearing the Lsb2 prion in the Ssb-deficient culture after heat stress (Figure 4B).

While the Hsp70-Ssb protein is specific to fungi, its RAC cochaperones are conserved from yeast to mammals, so that other member (s) of the Hsp70 family play roles similar to Ssb in mammalian cells [49]. Indeed, human orthologs of Zuo1 and Ssz1 are shown to antagonize [*PSI*^+^] formation in yeast cells lacking endogenous RAC [50]. Therefore, the relationship between the ribosome-associated chaperone machinery and self-perpetuating protein aggregation established in our work could likely be relevant beyond yeast and may contribute to both protein assembly disorders and biological effects of self-perpetuating protein aggregation in higher eukaryotes.

## 4. Materials and Methods

### 4.1. Yeast Strains

The *S. cerevisiae* strains used in this study are listed in Table S15. The isogenic haploid *MAT***a** [*psi^−^ pin*^−^] yeast strains with (GT1786, [27]) or without (GT409, [40]) *ssb1/2Δ* deletion and [*psi^−^ PIN*^+^] strains with (GT157) or without (GT159) *ssb1/2Δ* deletion [23] derived from the GT81 series were described earlier. All [*LSB*^+^], [*PRN*^+^], and [*STE*^+^] isolates were obtained in the [*psi^−^ pin*^−^] strains, while experiments checking the impact of *ssb1/2Δ* on the mitotic stability of newly formed [*PSI*^+^] isolates were performed in the [*psi^−^ PIN*^+^] strains. The isogenic WT [*psi^−^ pin*^−^] strain of the opposite mating type, *MATα* (GT197), was described previously [40]. The isogenic [*PSI^+^ PIN*^+^] *MAT**α*** strain GT1780-1D bearing *ssb1/2Δ* was constructed by D. Kiktev via mating haploid strains of the GT81 origin with *zuo1Δ::HIS3* and with *ssb1Δ::HIS3* and *ssb2Δ::ura3* deletions, followed by sporulating and dissecting resulting diploid, and verifying deletion combinations in the spore clones by PCR. The [*psi^−^ pin*^−^] *MAT**α** ssb1/2Δ* strain GT2340 was produced via curing the [*PSI*^+^] and [*PIN*^+^] prions from the strain GT1780-1D (described above) by GuHCl. The [*psi^−^ pin*^−^] *MATα* strains were used in genetic crosses to reintroduce chaperones Ssb or Zuo1, to introduce plasmids overexpressing Hsp104 or to produce dominant negative derivative of Hsp104. WTY664 is an *lsb2Δ* derivative of the [*psi^−^ pin*^−^] strain GT409, constructed as described previously [31]. GT2383 is an *lsb2Δ* derivative of the [*psi^−^ pin*^−^] *ssb1/2Δ* strain GT1786, constructed by replacing the *LSB2* gene with the bacterial *KanMX* gene, conferring resistance to G418 and PCR-amplified from the pFA6a-KanMX6 plasmid as in ref. [51]. The [*ure3-0*] strain BY241 [52] was kindly provided by R. Wickner. The *zuo1Δ* (GT2175) derivative of BY241 was constructed by replacing the *ZUO1* gene with the *KanMX* gene as in ref. [51]. The *ssb1/2Δ* (GT2438) derivative of BY241 was constructed by two subsequent replacements of the *SSB1* and *SSB2* genes with *TRP1* and *URA3* genes, respectively, PCR-amplified from respective plasmids [51] using primers with extensions homologous to flanking region of a respective gene.

### 4.2. Plasmids

Basic centromeric *URA3* yeast vector pRS316 [53] was typically used as an empty control for *URA3*-based plasmids. Centromeric *URA3* plasmids, bearing the WT or HA-tagged *LSB2*, mutant derivatives of *HA-LSB2* and the *LSB2-GFP* construct [10,31,54], or *HA-STE18* and *GFP-STE18* constructs [13] under the control of the copper-inducible *P_CUP1_* promoter, were described earlier. The centromeric *TRP1* plasmid pFL39GAL-SUP35N [55], containing region coding for the first 113 amino acid residues of the Sup35N domain under the control of the galactose-inducible *P_GAL_* promoter, was used to induce [*PSI*^+^] formation in the [*psi*^−^] strains. Centromeric *URA3* plasmids pRS316-TEF-SSB1 [40] and pRS416-TEF-ZUO1 [56], bearing the *SSB1* and *ZUO1* genes, respectively, under the control of the translation elongation factor 1α promoter (*P_TEF_*) were kindly provided by E. Craig. Multicopy 2µ DNA-based *LEU2* plasmid YEp351-GDP-GFP-SSB1, bearing the *GFP-SSB1* construct under the *P_GPD_* promoter, was created by cloning the *Sac*I-*Not*I fragment, containing the chimeric *GFP-SSB1* ORF from the plasmid pRS316-GFP-SSB1 [57], kindly provided by E. Deuerling, into the plasmid pRS316-GPD [58], cut with the same enzymes, and then transferring the *Sal*I-*Sac*I fragment, containing the *P_GDP_-GFP-SSB1* cassette, from this plasmid to YEp351 [59] cut with the same enzymes. Centromeric *LEU2* plasmid p366-SSB2, bearing the *SSB2* gene under its own promoter, was identified by J. Patterson in Chernoff lab from the p366-based yeast genomic library, kindly provided by P. Hieter. The centromeric plasmids bearing the WT *HSP104* gene under the highly expressed *P_GPD_* promoter and *LEU2* marker, pLH105, or the dominant negative allele of the *HSP104*, *HSP104-DN* (with double K218,620T substitution inactivating both ATP-binding sites), and *URA3* marker [60], originating from the laboratory of S. Lindquist, were based on pRS415 [53] and pRS316, respectively.

### 4.3. Growth Conditions and Phenotype Detection

Standard protocols were used for the preparation of the yeast complete organic (YPD) and synthetic media and for yeast transformation [61]. The synthetic yeast medium contained 3 μM copper sulfate (CuSO_4_); it was supplemented with 25 to 200 μM CuSO_4_ (as indicated) in order to increase expression of genes placed under the copper promoter (*P_CUP1_*). Typically, 2% glucose was used as a carbon source. However, 2% galactose was added instead of glucose to induce genes placed under a galactose-inducible promoter (*P_GAL_*). 5-fluoroorotic acid (5-FOA) was used in screens to select against *URA3* plasmids where necessary [62]. Organic YPG medium containing 3% glycerol instead of glucose was used to identify respiratory-incompetent (Pet^−^) colonies, arising from mitochondrial DNA loss, either spontaneously or during transformation. Due to their slow growth and inability to induce *P_GAL_*, their colonies were hard to employ in some of our assays; thus, they were typically excluded from analysis. Yeast cells were incubated at 30 °C unless stated otherwise. Samples of 10 or 50 mL placed into Oakridge 25 mL round-bottom tubes and 250 mL Erlenmeyer flasks, respectively, to grow yeast cultures in the liquid medium with shaking at 200 rpm. The optical density of growing yeast cultures was monitored at 600 nm (OD_600_) using a Shimadzu UV-2450 spectrophotometer. The presence of [*PSI*^+^] or [*URE3*] was determined by growth on –Ade medium and a lighter color on YPD medium, which originated either from readthrough of the UGA reporter allele *ade1-14* [4] or from induction of the [*URE3*]-dependent *P_DAL5_*-*ADE2* reporter construct [39], respectively. It should be noted that non-prion ([*psi*^−^] or [*ure3-0*]) Ade^−^ strains with *ssb1/2Δ* or *zuo1Δ* deletions typically exhibit a somewhat lighter color on YPD medium, compared to their Ssb^+^ and Zuo^+^ counterparts. This phenomenon is apparently due to partly impaired accumulation of red pigment in the Ssb- or RAC-deficient strains; however, it does not prevent color differentiation between the non-prion and prion cultures in respective backgrounds. The presence of [*LSB*^+^] or [*STE*^+^] was detected phenotypically by their ability to promote de novo formation of [*PSI*^+^] after transient overproduction of Sup35N [10,13]. For this purpose, the cultures bearing the *ade1-14* (UGA) reporter and plasmid pFL39GAL-SUP35N were incubated on the galactose medium selective for the plasmid, where the *P_GAL_-SUP35N* construct is induced, and then velveteen-replica-plated onto the glucose –Ade medium for [*PSI*^+^] detection. Excess Sup35N efficiently induces [*PSI*^+^] formation only in the presence of other protein aggregates [4]. To assess prion curing by guanidine hydrochloride (GuHCl), an inhibitor of the chaperone Hsp104 [35,63], yeast cultures were grown in parallel on the solid YPD medium and on YPD containing 5 mM (for the WT strains) or 2 mM (for the *ssb1/2Δ* or *zuo1Δ* strains that are hypersensitive to GuHCl) of GuHCl for three passages (typically 20–40 generations), followed by streak outs on the medium lacking GuHCl and testing individual subcolonies (usually at least 8 or more from each original culture) for the presence of a respective prion using the phenotypic assays described above.

### 4.4. Spontaneous Formation of the [URE3] Prion

To measure the frequencies and rates of the spontaneous formation of [*URE3*], a fluctuation assay was performed in the same way as described previously for [*PSI*^+^] [23]. No less than 12 independent cultures were analyzed for each strain. The frequency of Ade^+^ colonies was calculated as the ratio of Ade^+^ colonies to the total number of colony-forming units (viable cells) plated. The rate (R) of Ade^+^ colonies (reflecting the rate of spontaneous [*URE3*] formation) was calculated according to the formula *R* = *f*/*ln(NR),* where *f* is the observed frequency of Ade^+^ colonies and *N* is the number of cells in the culture. Median rates and 95% confidence limits were determined according to standard formulas [64]. To confirm that the majority of Ade^+^ colonies indeed originated from prion formation rather than from chromosomal mutation, tests for mitotic stability and curing by GuHCl were performed.

### 4.5. Prion induction by Heat Shock

To monitor the formation of prions with [*PSI*^+^]-inducing capabilities during mild heat stress, the WT and *ssb1/2Δ* [*pin^−^ psi*^−^] yeast strains bearing a plasmid with the *P_GAL_-SUP35N TRP1* construct were grown in the liquid YPD medium overnight at 25 °C, inoculated into fresh YPD with starting OD_600_ at about 0.1, and incubated for 2 h at 25 °C with shaking at 200 rpm, followed by incubation for 2 h at 39 °C. The samples taken before and after 39 °C treatment were plated onto the glucose medium selective for the plasmid (-Trp) at a density of 200–300 cells per plate and grown at 25 °C for 3–7 days. Grown colonies were velveteen-replica-plated to the same medium with glucose (-Trp) or with galactose (-Trp + Gal) and incubated for 2 days, followed by velveteen-replica-plating to -Ade medium. Colonies that produced growth or extensive papillation on -Ade media after -Trp + Gal (but not after -Trp) were considered prion-containing colonies, capable of [*PSI*^+^] induction after Sup35N overproduction. A subset of Ade^+^ subcolonies were passaged on GuHCl to check for curability, thus confirming that growth on -Ade was indeed due to the formation of [*PSI*^+^] prion.

### 4.6. Mating Assays for [LSB^+^] Retention

To check the effects of Hsp104 inactivation or overproduction on [*LSB*^+^] prions in the absence of Ssb, the *ssb1/2Δ* [*LSB*^+^] isolates that lost the *P_CUP1_-HA-LSB2* plasmid but retained the *TRP1 P_GAL_-SUP35N* construct and control *ssb1/2Δ* [*lsb*^−^] isolates bearing *P_GAL_-SUP35N* were mated to the *ssb1/2Δ* [*lsb*^−^] strain of the opposite mating type, bearing the following *URA3* plasmids: empty vector; dominant negative allele of *HSP104* (*HSP104-DN*); or *WT HSP104* overexpressor cassette under the *P_GPD_* promoter. In order to check the effects of Ssb reintroduction on [*LSB*^+^] prions obtained in the absence of Ssb, both *MAT***a** *ssb1/2Δ* [*LSB*^+^] isolates that lost the *P_CUP1_-HA-LSB2* plasmid but retained the *TRP1 P_GAL_-SUP35N* construct, as well as control *ssb1/2Δ* [*lsb*^−^] isolates bearing *P_GAL_-SUP35N*, were mated either to the isogenic *MATα* [*psi^−^ pin^−^ lsb*^−^] *Ssb*^+^ strain or to the isogenic *MATα* [*psi^−^ pin^−^ lsb*^−^] *ssb1/2Δ* strain, carrying either the control *URA3* vector or the *URA3* plasmid with the constitutively expressed *P_TEF_-SSB1* construct. For all selected diploids containing *URA3* plasmids, these plasmids were cured by counterselection on 5-FOA medium. Then, Sup35N was induced on galactose medium, followed by detection of [*LSB*^+^] prion via its ability to cross-seed the formation of [*PSI*^+^] prion, as seen on -Ade medium.

### 4.7. Protein Analysis

For protein isolation, yeast cells were collected by centrifugation at 3000 rpm for 5 min at 4 °C, resuspended in 100–300 μL of ice-cold lysis buffer (25 mM Tris pH 7.5, 0.1 M NaCl, 10 mM EDTA, 100 μg/mL cycloheximide, 2 mM benzamidine, 20 μg/mL leupeptin, 4 μg/mL pepstatin A, 1 mM N-ethylmaleimide, 1× protease inhibitor cocktail from Roche Diagnostics, 2 mM PMSF), mixed with ¼ (volume/volume) of 150–212 µM acid washed glass beads from Sigma-Aldrich (Burlington, MA, USA, catalog #G1145), and disrupted by vortexing at 4 °C for 6 min. After removing cell debris by centrifugation at 5900 g for 2 min, the supernatant was transferred to a clean microcentrifuge tube, and protein concentrations were measured using the colorimetric Bradford protein assay (BIO-RAD) [65]. Proteins were fractionated by sodium dodecyl sulfate–polyacrylamide gel electrophoresis (SDS-PAGE) and identified by Western blotting analysis followed by reaction to respective antibodies. For identification of detergent-resistant protein aggregates, either “boiled gel” SDS-PAGE or semi-denaturing detergent-agarose gel electrophoresis (SDD-AGE) were employed [34,66]. In a “boiled gel” assay, SDS-containing protein samples (either pre-boiled as a control or not pre-boiled) were run on the SDS-PAGE gel for about 1 h, followed by interrupting electrophoresis, sealing wells with additional polyacrylamide, and “boiling” the whole gel within the plastic bag in the steamed water bath for 10 min. After boiling, the gel was placed into the electrophoretic apparatus and electrophoresis was resumed. Detergent-resistant prion polymers are unable to enter the SDS-PAGE without boiling; however, they are solubilized and enter the gel after boiling. In SDD-AGE, aggregates and monomers from a sample containing a detergent (either 3% sodium N-lauroylsarkosine or 2% SDS) are separated by electrophoresis in the agarose gel with buffer, containing 0.1% SDS, roughly in accordance with their sizes. After reaction to primary and respective secondary antibodies, proteins were visualized using the chemiluminescent detection reagent from GE HealthCare (Chicago, IL, USA) as described in the GE HealthCare protocol. Rabbit polyclonal antibodies to Ade2, Ssb, and Rnq1 were kindly provided by Dr. V. Alenin, Dr. E. Craig, and Dr. S. Lindquist, respectively. Rabbit antibodies to HA (C29F4) and G6PDH (catalog #A9521) were obtained from Cell Signaling Technology (Danvers, MA, USA) and Sigma-Aldrich, respectively. Rabbit antibodies recognizing both Lsb1 and Lsb2 were generated by ProSci, Inc. (Poway, CA, USA) and described previously [31]. These antibodies recognize Lsb2 and both full-length and heat-shock-induced proteolytically processed (shortened) forms of its paralog Lsb1 [54]. Densitometry was performed using the program ImageJ, National Institutes of Health (Bethesda, MD, USA), downloaded on 20 August 2021 from https://imagej.nih.gov. Levels of proteins measured (such as Lsb2 and its derivatives and Ste18) were always normalized by either Ade2 or G6PDH, which were used as loading controls.

### 4.8. Fluorescence Microscopy

To visualize protein aggregates formed by Lsb2 or Ste18 in vivo, these proteins were tagged by the green fluorescent protein (GFP) fluorophore. Cultures of strains bearing the *LSB2-GFP* or *GFP-STE18* cassette on a plasmid under the control of the copper-inducible (*P_CUP1_*) promoter were grown overnight in the liquid media selective for a plasmid (-Ura) and inoculated each into 10 mL of the same fresh medium either not containing or containing extra CuSO_4_ (as specified in Figure 1C and Figure 5C), with a starting OD_600_ of 0.4. 500 μL aliquots, which were taken from each culture at specified time points; cells were spun down at 3500 rpm for 2 min, then 10 μL aliquot of each sample was taken from the bottom of the tube, placed onto a microscope slide with cover slip, and sealed with clear nail polish to prevent drying. Fluorescence was monitored using a BX41 microscope (Olympus, Center Valley, PA, USA) at 100× (oil immersion) with the endow GFP bandpass emission (green) filter. Images were taken using an Olympus DP-71 camera.

## 5. Conclusions

Our data demonstrate that the ribosome-associated chaperone Hsp70-Ssb antagonizes formation and/or heritability of prion aggregates formed by a variety of yeast proteins, such as Lsb2, Ste18, and Ure2 (in addition to Sup35, for which this effect has been demonstrated previously). The Hsp40 cochaperone of Ssb, Zuo1 also influences the formation and propagation of the prion form of Ure2. Almost 20% of the cells form a detectable prion after mild heat stress in yeast culture lacking Ssb. The majority of these stress-induced prions represent a prion form of the Lsb2 protein. These results implicate Hsp70-Ssb as a major modulator of heritable protein aggregation and stress memory with a broad spectrum of action.

## Data Availability

The data presented in this study are available in the article and supporting information.

## Supplementary Materials

Supporting information can be downloaded at: https://www.mdpi.com/article/10.3390/ijms24108660/s1.
