# Supplementary material for: Yeast Chaperone Hsp70-Ssb Modulates a Variety of Protein-Based Heritable Elements
Source: Int J Mol Sci. 2023 May 12;24(10):8660. doi: 10.3390/ijms24108660 (PMC10218661; doi:10.3390/ijms24108660)
Supplement: Supplementary file 1 [file ijms-24-08660-s001.zip › ijms-2366647-supplementary.pdf]

Yeast chaperone Hsp70-Ssb modulates a variety of protein-based heritable elements

Lina M. Jay-Garcia, Joseph L. Cornell, Rebecca L. Howie, Quincy L. Faber, Abigail Salas, Tatiana A. Chernova, and Yury O. Chernoff*

SUPPLEMENTAL MATERIALS

List of materials

Supplemental Figures, S1 through S3

Supplemental Tables, S1 through S15

Supplemental Figures

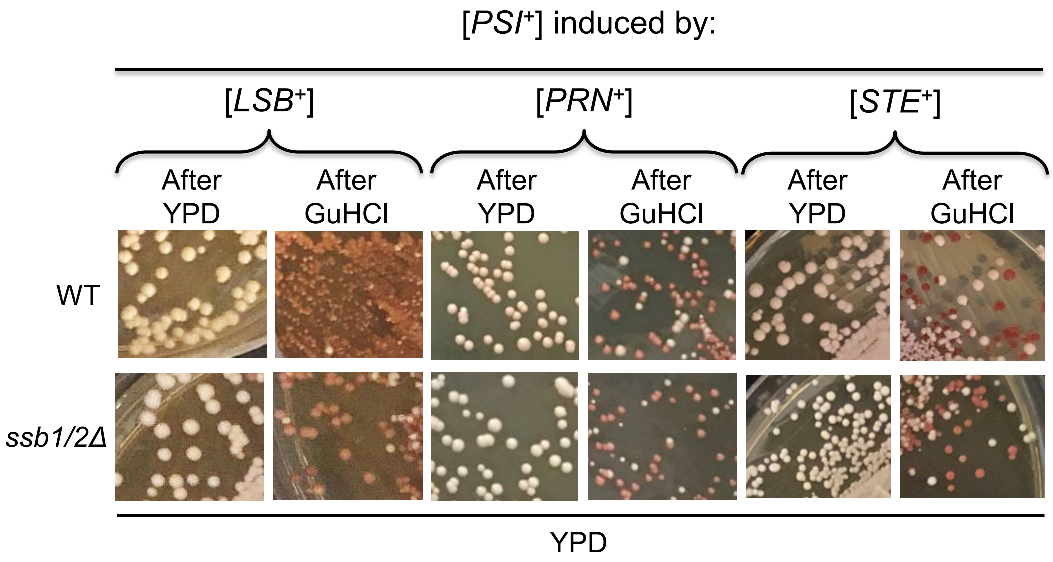

Figure S1. Curing of [*PSI*+] colonies induced in the presence of various prions by growth in the presence of GuHCl. Colonies were passaged three times on either YPD medium or YPD medium with GuHCl (5 mM for WT and 2 mM for *ssb1/2Δ*), and then streaked out for single subcolonies on a fresh YPD plate. [*PSI*+] loss is seen by appearance of red and mosaic colonies. Typical examples are shown. See Table S5 for numbers.

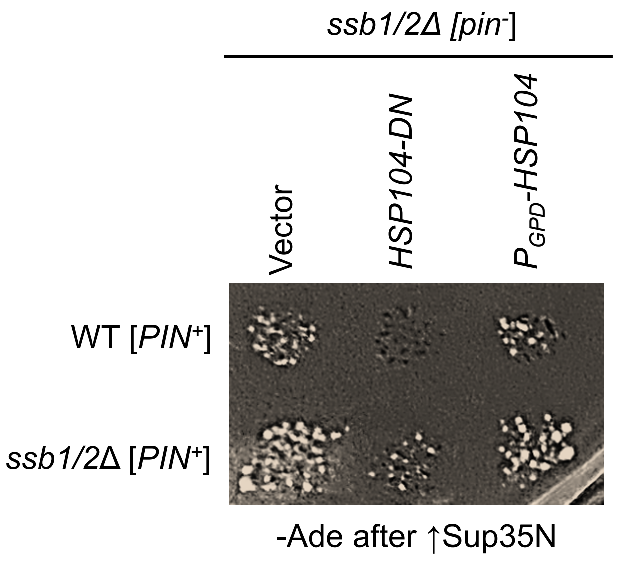

Figure S2. Curing of [*PIN*⁺] prion by transient inactivation of Hsp104. The experiment was performed by mating assay in the same as described in Materials and Methods and shown on Fig. 2E for [*LSB*⁺]. The wild-type (WT) and *ssb1/2Δ* [*PIN*⁺] isolates containing the *TRP1* P_{GAL}-*SUP35N* construct were mated to the *ssb1/2Δ* [*pin*⁻] strain of opposite mating type, bearing the following *URA3* plasmids: empty vector (Vector), dominant negative allele of *HSP104* (*HSP104-DN*), or *WT HSP104* overexpressor cassette (P_{GPD}-*HSP104*) under the P_{GPD} promoter. After the selection of diploids on –Ura-Trp, the [*URA3*] plasmids were cured by counterselection on 5-FOA medium. Then, Sup35N was induced on galactose medium, followed by detection of [*PIN*⁺] prion via its ability to cross-seed the formation of [*PSI*⁺] prion, as seen on –Ade medium. Transient overproduction of Hsp104-DN (but not transient overproduction of wild-type Hsp104) cures most cells of [*PIN*⁺], resulting in a great decrease in [*PSI*⁺] formation.

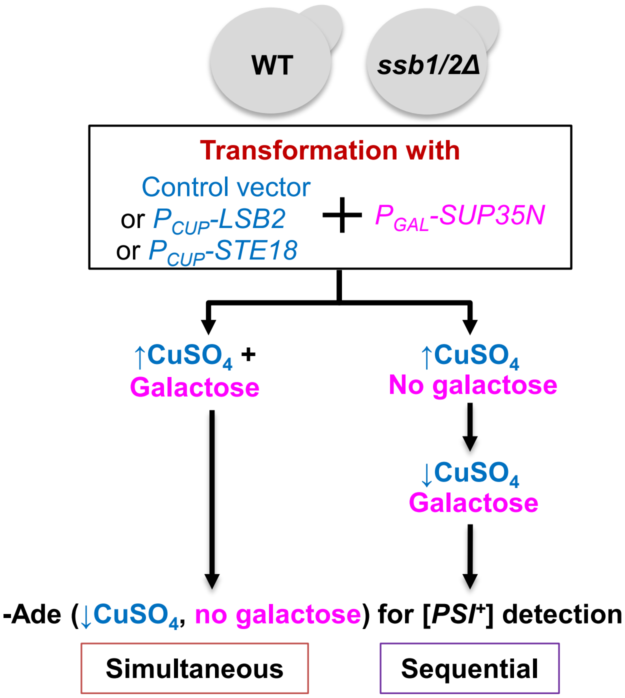

Figure S3. Simultaneous and sequential overproduction protocols for [*PSI*$^+$] induction. In the simultaneous overproduction protocol, both *LSB2* (wild-type or mutant) or *STE18* construct (under P_{CUP1} promoter) and *SUP35N* construct (under the P_{GAL} promoter) are overexpressed simultaneously on the medium containing both extra $CuSO_4$ (the inducer of P_{CUP1}) and galactose (the inducer of P_{GAL}). In the sequential overproduction protocol, a construct under the P_{CUP1} promoter is overexpressed first in the conditions when a construct under the P_{GAL} promoter is silent. (The control plate without overexpression is run in parallel.) This is followed by overexpression of a P_{GAL} construct in the conditions when overexpression of a P_{CUP1} construct is turned off. Following overexpression, the plates are velveteen replica plated to -Ade medium (lacking both extra $CuSO_4$ and galactose) for [*PSI*$^+$] detection. As experiments are performed with proliferating cultures, only heritable derivatives of [*LSB*$^+$] or [*STE*$^+$] should be capable of [*PSI*$^+$] induction in the sequential protocol.

Supplemental Tables

Table S1. Aggregation of Lsb2 as detected by fluorescence microscopy.

Strains	Plasmid	Time (h)	No extra $CuSO_4$				100 µM $CuSO_4$			
			Cells with aggregates			Total*	Cells with aggregates			Total*
			Number	%	SE**		Number	%	SE**	
WT	*P_{CUP1}-GFP*	0	0	0.0	0.6	152	0	0.0	0.6	152
		6	0	0.0	0.4	188	0	0.0	0.2	255
		12	0	0.0	0.4	221	0	0.0	0.4	207
		24	0	0.0	0.3	263	0	0.0	0.5	197
		48	0	0.0	0.5	201	0	0.0	0.3	183
WT	*P_{CUP1}-LSB2-GFP*	0	6	1.7	1.7	260	6	1.7	1.7	260
		6	3	2.5	1.3	166	53	32.2	5.1	167
		12	1	1.8	1.8	60	17	22.1	5.2	82
		24	0	0.0	0.3	308	27	4.8	4.8	357
		48	0	0.0	0.5	208	0	0.0	0.3	298
ssb1/2Δ	*P_{CUP1}-GFP*	0	0	0.0	0.4	253	0	0.0	0.4	253
		6	0	0.0	0.6	143	0	0.0	0.4	191
		12	0	0.0	0.7	127	0	0.0	0.4	214
		24	0	0.0	0.4	248	0	0.0	0.4	205
		48	0	0.0	0.3	260	0	0.0	0.4	216
ssb1/2Δ	*P_{CUP1}-LSB2-GFP*	0	12	8.8	4.1	160	12	8.8	4.1	160
		6	2	13.3	3.3	16	34	43.9	3.4	78
		12	7	5.4	1.9	126	10	24.4	0.5	41
		24	24	7.8	5.7	434	55	13.7	1.3	380
		48	5	1.9	0.6	255	15	3.3	3.3	272

* Cells with fluorescence.
**SE refers to standard error.

Data for Fig. 1, C and D.

Table S2. Effect of *ssb1/2Δ* on [*LSB2*$^+$] induction by Lsb2 overexpression.

Strains	Plasmid-based construct	Number of cultures analyzed	[*PSI*$^+$]-inducing colonies			Total colonies
			Number	%	SD*	
WT	P_{CUP1}	4	0	0.0	0.1	965
	P_{CUP1}-*HA-LSB2*	6	16	0.8	1.0	2019
ssb1/2Δ	P_{CUP1}	4	23	4.3	1.9	538
	P_{CUP1}-*HA-LSB2*	6	255	18.6	2.9	1372

Data for Fig. 1, E and F.
*SD refers to standard deviation.

Table S3. Mitotic stability of the [*LSB*$^+$] and [*PRN*$^+$] prions in the WT and *ssb1/2Δ* backgrounds.

Prion	Induced by	Strain	Mitotic stability*			Total tested
			High	Intermediate	Low	
[*LSB*$^+$]	↑Lsb2	WT	0	1<br>(18%)	3<br>(0-9%)	4
		ssb1/2Δ	5<br>(40-72%)	0	0	5
	↑Lsb2<br>(tested after the loss of *LSB2* plasmid)	*ssb1/2Δ*	4<br>(37.5-67%)	0	2<br>(0-8%)	6
[*PRN*$^+$]	39°C	WT	0	0	2	2
		ssb1/2Δ	0	8<br>(12-28%)	4<br>(0-6%)	12

Examples are shown on Figs. 2A and 6C.
*The observed ranges of prion retention for a given category are indicated in parenthesis.

Table S4. Curability of various prions by GuHCl in the *ssb1/2Δ* background.

Prion	Plasmid-based construct	Curable	Incurable	Unstable undetermined	Total
[*LSB*$^+$]	P_{CUP1}-*HA-LSB2*	0	5	0	5
	None	0	3	2	5
[*PRN*$^+$]	None	0	8	4	12
[*STE*$^+$]	P_{CUP1}-*HA-STE18*	0	7	2	9
	None	0	2	2	4

Examples are shown on Figs. 2C, 6D and 8E.

Table S5. Effect of GuHCl on the [*PSI*$^+$] isolates induced in the presence of various prions.

[*PSI*$^+$] isolates induced by	Strain	Curable	Incurable	Total
[*LSB*$^+$]	WT	6	0	6
	ssb1/2Δ	12	0	12
[*PRN*$^+$]	WT	3	0	3
	ssb1/2Δ	12	0	12
[*STE*$^+$]	*ssb1/2Δ*	6	0	6

Examples are shown on Fig. S1.

Table S6. Formation of [*PRN*$^+$] prions during heat stress.

Strains	Number of cultures analyzed	[*PSI*$^+$]-inducing colonies before heat shock			[*PSI*$^+$]-inducing colonies after 2 hrs at 39°C		
		Number	% (± SD*)	Total colonies tested	Number	% (± SD*)	Total colonies tested
WT	6	5	0.2 ± 0.1	3437	10	1.2 ± 1.0	4184
ssb1/2Δ	6	119	6.3 ± 1.5	11698	676	16.9 ± 1.5	3825
ssb1/2Δ lsb2Δ	6	7	1.0 ± 0.5	1797	14	1.9 ± 0.9	1680

Data for Fig. 4, A and B.
*SD refers to standard deviation.

Table S7. Aggregation of Ste18 as detected by fluorescence microscopy.

Strains	Plasmid-based construct	Time (h)	No extra $CuSO_4$				150 μM $CuSO_4$			
			Cells with aggregates			Total*	Cells with aggregates			Total*
			#	%	SE**		#	%	SE**	
WT	P_{CUP1}-*GFP*	0	0	0	0.3	377	0	0	0.3	377
		6	0	0	0.2	388	0	0	0.3	371
		24	0	0	0.2	413	0	0	0.2	471
	P_{CUP1}-*GFP-STE18*	0	0	0	0.1	743	0	0	0.1	743
		6	0	0	0.2	548	5	2.3	2.7	486
		24	0	0	0.1	721	0	0	0.1	748
ssb1/2Δ	P_{CUP1}-*GFP*	0	0	0	0.2	557	0	0	0.2	557
		6	0	0	0.2	417	0	0	0.2	392
		24	0	0	0.2	511	0	0	0.3	433
	P_{CUP1}-*GFP-STE18*	0	0	0	0.2	617	0	0	0.2	617
		6	11	2.8	1.6	489	46	8.1	2.4	536
		24	0	0	0.1	762	2	0.6	0.7	613

*Cells with fluorescence.
**SE refers to standard error.
Data for Fig. 5, C and D.

Table S8. Effect of *ssb1/2Δ* on the formation of [*STE*$^+$].

Strains	Plasmid-based construct	Number of cultures analyzed	Number of inducible colonies	Frequency of inducible colonies		Total colonies
				%	SE*	
WT	P_{CUP1}	3	0	0	0.1	1092
	P_{CUP1}-*HA-STE18*	4	13	0.9	0.4	1421
ssb1/2Δ	P_{CUP1}	3	20	1.9	0.1	1073
	P_{CUP1}-*HA-STE18*	4	84	10.0	0.3	842

Data for Fig. 6, B and C.
*SE refers to standard error.

Table S9. Mitotic stability of [*STE*$^+$].

Origin	Strain	Mitotic stability*			Total
		High	Intermediate	Low	
Original isolates	WT	0	0	2 (0-7%)	2
	ssb1/2Δ	4 (33-58%)	4 (16-29%)	1 (8.5%)	9
Isolates after storage at -80°C	*ssb1/2Δ*	1 (75%)	2 (13-16%)	6 (0-8%)	9
Isolates after the loss of *STE18* plasmid	*ssb1/2Δ*	2 (36-45.5%)	4 (16-24%)	4 (0%)	10

Examples are shown on Fig. 6A.
*The observed ranges of prion retention for a given category are indicated in parentheses.

Table S10. Frequencies and rates of spontaneous [*URE3*] formation.

Strains	Number of cultures analyzed	Median frequency		Median rate	
		Ade^+	95% confidence limits	Ade^+	95% confidence limits
WT	30	$7.8x10^{-6}$	$(5.9\text{-}9.7)x10^{-6}$	$1.8x10^{-06}$	$(1.4\text{-}2.2)x10^{-6}$
ssb1/2Δ	30	$6.6x10^{-6}$	$(3.5\text{-}9.7)x10^{-6}$	$1.4x10^{-06}$	$(0.8\text{-}2.1)x10^{-6}$
zuo1Δ	18	$4.9x10^{-5}$	$(3.8\text{-}6.0)x10^{-5}$	$9.1x10^{-6}$	$(7.2\text{-}11.0)x10^{-6}$

Data for Fig. 7, A and B.

Table S11. Mitotic stability of the [*URE3*] prion isolates.

Strain	Mitotic stability*					% unstable	Total Ade^+ tested
	Unstable (0-10%)	Low stability (11-30%)	Intermediate stability (31-79%)	High stability (80-99%)	Completely stable (100%)		
WT	20	7	11	11	15	31.3	64
ssb1/2Δ	0	6	16	7	34	0.0	63
zuo1Δ	12	4	18	28	25	13.8	87

Data for Fig. 7, C and D.
*The observed ranges of prion retention for a given category are indicated in parentheses.

Table S12. Curability of the [*URE3*] prion isolates by GuHCl.

Strain	Intermediate stability		High stability		Completely stable		% Curable	Total colonies tested
	Curable	Incurable	Curable	Incurable	Curable	Incurable		
WT	6	2	10	0	12	7	75.7	37
ssb1/2Δ	12	5	11	0	4	2	79.4	34
zuo1Δ	14	10	10	4	5	3	63.0	46

Note: Highly unstable [*URE3*] prion isolates were excluded from curability assays.

Table S13. Effect of the Ssb reintroduction on [*URE3*] isolates obtained in the *ssb1/2Δ* background.

Strain	Colony	Prion manifestation after reintroduction Ssb				Prion mitotic stability after reintroduction of Ssb	
		SSB1 (*P_{GDP}*- *SSB1*)		*SSB2* (*P_{SSB2}*- *SSB2*)		*SSB1* (*P_{GDP}*- *SSB1*)	*SSB2* (*P_{SSB2}*- *SSB2*)
		Retention* of [*URE3*]	Sensitivity** of [*URE3*]	Retention* of [*URE3*]	Sensitivity** of [*URE3*]	Stability of [*URE3*]	Stability of [*URE3*]
WT	1	Yes	I	Yes	I	U	U
	2	Yes	I	Yes	I	U	U
	3	Yes	I	Yes	I	U	St
	4	Yes	I	Yes	I	U	U
	5	Yes	I	Yes	I	U	NT
	6	Yes	I	Yes	I	U	NT
ssb1/2Δ	1	Yes	PS	No	S	D	D
	2	Yes	PS	NT	NT	D	NT
	3	Yes	PS	Yes	PS	D	D
	4	No	S	NT	NT	NA	NT
	5	Yes	PS	Yes	PS	D	D
	6	Yes	PS	Yes	I	D	U
	7	Yes	PS	Yes	PS	D	D
	8	Yes	PS	No	S	D	D
	9	No	S	NT	NT	NA	NT

*As determined by a comparison between –Ade and –Ade-Leu medium.
**As detected by growth on –Ade-Leu medium and color on complete medium.

Designations.
Prion manifestation: S – sensitive, PS - partially sensitive, I - insensitive.
Mitotic stability: D – destabilized, U - unchanged, St – stabilized.
NT - not tested, NA – not applicable.
Examples of plate images are shown on Fig. 7E.

Table S14. Effect of Zuo1 reintroduction on [*URE3*] isolates obtained in the *zuo1Δ* background.

Strain	Colony*	Growth after reintroduction Zuo1 (P_{TEF}- *ZUO1*)		Destabilization after reintroduction Zuo1
		Retention of [*URE3*]	Sensitivity of [*URE3*]	Stability of [*URE3*]
WT	1	Yes	I	U
	2	Yes	I	U
	3	Yes	I	U
	4	Yes	I	U
	5	Yes	I	St
	6	Yes	I	U
zuo1Δ	1	No	S	NA
	2	No	S	NA
	3	Yes	I	U
	4	Yes	I	U

*Only [*URE3*] isolates curable by GuHCl were checked.
Designations are the same as for Table S13.
Plate images are shown on Fig. 7F.

Table S15. Yeast strains.

Name	Prion background	Genotype or origin
GT409	[*psi⁻ pin⁻*]	*MAT**a** ade1-14 his3 leu2-3,112 lys2 trp1 ura3-52*
GT197	[*psi⁻ pin⁻*]	GT409 *MATα*
GT1786	[*psi⁻ pin⁻*]	GT409 *ssb1Δ::HIS3 ssb2Δ::URA3*
GT2340	[*psi⁻ pin⁻*]	*MATα ade1-14 his3 leu2-3,112 lys2 trp1 ura3-52 ssb1Δ::HIS3 ssb2Δ::ura3*
GT2283	[*psi⁻ pin⁻*]	GT1786 *lsb2Δ::kanMX4*
WTY664	[*psi⁻ pin⁻*]	GT409 *lsb2Δ::kanMX4*
GT159	[*psi⁻ PIN⁺*]	*MAT**a** ade1-14 his3 leu2-3,112 lys2 trp1 ura3-52*
GT157	[*psi⁻ PIN⁺*]	GT159 *ssb1Δ::HIS3 ssb2Δ::URA3*
BY241	[*ure3-0*]	*MAT**a** leu2 trp1 ura3 P_{DAL5}::ADE2 P_{DAL5}::CAN1 kar1-1*
GT2175	[*ure3-0*]	BY241 *zuo1Δ::kanMX4*
GT2398	[*ure3-0*]	BY241 *ssb1Δ::TRP1*
GT2438	[*ure3-0*]	BY241 *ssb1Δ::TRP1 ssb2Δ::URA3*
